# AMPK phosphorylation of FNIP1 (S220) controls mitochondrial function and muscle fuel utilization during exercise

**DOI:** 10.1126/sciadv.adj2752

**Published:** 2024-02-07

**Authors:** Liwei Xiao, Yujing Yin, Zongchao Sun, Jing Liu, Yuhuan Jia, Likun Yang, Yan Mao, Shujun Peng, Zhifu Xie, Lei Fang, Jingya Li, Xiaoduo Xie, Zhenji Gan

**Affiliations:** ^1^ State Key Laboratory of Pharmaceutical Biotechnology and MOE Key Laboratory of Model Animal for Disease Study, Model Animal Research Center, Division of Spine Surgery, Department of Orthopedic Surgery, Nanjing Drum Tower Hospital, The Affiliated Hospital of Nanjing University Medical School, Jiangsu Key Laboratory of Molecular Medicine, Chemistry and Biomedicine Innovation Center (ChemBIC), Medical School of Nanjing University, Nanjing University, Nanjing, China.; ^2^ School of Medicine, Shenzhen Campus of Sun Yat-sen University, Sun Yat-sen University, Shenzhen, China.; ^3^ Shanghai Institute of Materia Medica, Chinese Academy of Sciences, Shanghai, China.; ^4^ Jiangsu Key Laboratory of Molecular Medicine & Chemistry and Biomedicine Innovation Center, Medical School of Nanjing University, Nanjing, China.

## Abstract

Exercise-induced activation of adenosine monophosphate–activated protein kinase (AMPK) and substrate phosphorylation modulate the metabolic capacity of mitochondria in skeletal muscle. However, the key effector(s) of AMPK and the regulatory mechanisms remain unclear. Here, we showed that AMPK phosphorylation of the folliculin interacting protein 1 (FNIP1) serine-220 (S220) controls mitochondrial function and muscle fuel utilization during exercise. Loss of FNIP1 in skeletal muscle resulted in increased mitochondrial content and augmented metabolic capacity, leading to enhanced exercise endurance in mice. Using skeletal muscle–specific nonphosphorylatable FNIP1 (S220A) and phosphomimic (S220D) transgenic mouse models as well as biochemical analysis in primary skeletal muscle cells, we demonstrated that exercise-induced FNIP1 (S220) phosphorylation by AMPK in muscle regulates mitochondrial electron transfer chain complex assembly, fuel utilization, and exercise performance without affecting mechanistic target of rapamycin complex 1–transcription factor EB signaling. Therefore, FNIP1 is a multifunctional AMPK effector for mitochondrial adaptation to exercise, implicating a mechanism for exercise tolerance in health and disease.

## Teaser

Exercise-induced AMPK-regulated FNIP1 S220 phosphorylation is critical for muscle mitochondrial metabolism and exercise capacity.

## INTRODUCTION

Physical exercise, as the cornerstone of a healthy human lifestyle, triggers cascades of physiological responses in the skeletal muscle, leading to enhanced energy expenditure and metabolic adaptations in all vertebrates (*1**,* *2*). Mitochondria, often referred to as the “powerhouses of the cell,” are the major cellular organelle that acts as an integrated signal platform to control cellular metabolism in muscle fibers for energy generation and metabolic adaptation during exercise (*3**,* *4*). The metabolic capacity and biogenesis of mitochondria are greatly promoted within cells as physical activity increases. One of the key mediators involved in this process is the adenosine monophosphate (AMP)–activated protein kinase (AMPK), an enzyme known as the cellular energy sensor or mitochondria guardian (*5**,* *6*). During exercise, the depletion of cellular energy, namely, adenosine triphosphate (ATP), and the subsequent increase in the AMP/ATP ratio activate AMPK, which phosphorylates effector proteins to promote glucose uptake and fatty acid oxidation, achieving a condition of energy balance and cell homeostasis (*5**–**7*). In addition to its well-known role in mediating mitochondrial metabolic adaptation via transcription factor–mediated mitochondrial biogenesis, AMPK also modulates mitochondrial activities through direct phosphorylation of multiple mitochondrial proteins to control oxidative phosphorylation (*8**–**10*). Drugs activate AMPK, act as exercise mimetics, and drive a mitochondrial oxidative program in the skeletal muscle (*11**–**13*). Exercise training activates AMPK and improves many chronic disease conditions through mitochondrial remodeling in the skeletal muscle (*3**,* *8**,* *14**,* *15*). Conversely, dysregulation of AMPK signaling and subsequent mitochondrial dysfunction in the skeletal muscle are frequently observed in the pathogenesis of human diseases, including obesity, type 2 diabetes, and muscular dystrophy/atrophy (*3**,* *4**,* *16**,* *17*).

Accumulated evidence indicates that AMPK mediates exercise-induced mitochondrial modulation through phosphorylation of key transcription factors such as transcription factor EB (TFEB) and peroxisome proliferation–activated receptor (PPAR)–γ coactivator 1α (PGC1α), which subsequently induces gene expression for mitochondrial biogenesis and quality control through coordination with estrogen-related receptors (ERRs) or PPAR nuclear receptors (*18**–**21*). In addition to de novo mitochondrial biogenesis, exercise is also effective in promoting favorable remodeling of the muscle mitochondrial proteome to coordinate the requirements of efficient mitochondrial biogenesis, reorganization, and assembly (*22**–**24*). For instance, mitochondrial electron transfer chain (ETC) respiratory supercomplex assemblies increase in response to exercise in the skeletal muscle (*22*). However, it remains unclear whether there are other AMPK substrates that are directly involved in mitochondrial ETC function, such as regulation of supercomplex assembly or regulation of ETC complex activity. Folliculin (FLCN) interacting protein 1 (FNIP1) is an adaptor protein originally identified as a binding partner for FLCN and AMPK (*25*). Previous studies have placed AMPK downstream of FLCN/FNIP1, which functional as a guanosine triphosphate–activating protein (GAP) for the RagC/D guanosine triphosphatase (GTPase) to sense amino acids in mechanistic target of rapamycin complex 1 (mTORC1) signaling (*26**–**29*). However, the exact roles of FLCN/FNIP1 in sensing energy status in the AMPK pathway or in sensing nutrients in mTOR signaling remain unclear.

Our previous studies indicated that *Fnip1*, as a target of the MyomiR *miR-499*, regulates mitochondrial function and muscle fiber type switching in an AMPK/PGC1α-dependent and -independent manner in myocytes and in skeletal muscle of mouse models (*30**–**32*). Consistently, FLCN or FNIP1 knockout mouse models have been shown to increase mitochondrial function and oxidative metabolism in the skeletal muscle, and FNIP1 also affects metabolic processes in other tissues (*26**,* *33**–**35*). Collectively, these studies suggest a general role for FNIP1 in metabolic regulation. Nevertheless, how FNIP1 mediates such a wide effect on metabolism and the exact relationship between FNIP1 and AMPK or mTORC1 signaling remain unclear. Deletion of FNIP1 led to both activation and inhibition of AMPK phosphorylation (*33**,* *34*), and, similarly, both suppression and activation of mTOR signaling by FNIP1 have also been reported (*29**,* *33**,* *34**,* *36*). One of the common effectors of AMPK and mTORC1 is TFEB, which mediates gene expression to control lysosome biogenesis and autophagy in response to nutrient deprivation and energetic stress (*18**,* *37*). AMPK and mTORC1 are known to directly phosphorylate TFEB at different sites with opposing effects on TFEB nuclear translocation and transcription (*19**,* *37**,* *38*). FNIP1 was also reported to be involved in this process (*18**,* *29**,* *39*), and it seems that there is complex reciprocal regulation among AMPK, FNIP1, and mTORC1 to control TFEB translocation.

Using muscle-specific gain- and loss-of-function mouse models, we investigated the physiological role of FNIP1 in mediating AMPK signaling in the skeletal muscle, and it was revealed that FNIP1 was directly phosphorylated by AMPK at S220 in response to exercise training, which appears to play an important role in the regulation of mitochondrial proteomics and related metabolic regulation, according to genetic and biochemical evidence. Notably, AMPK phosphorylation of FNIP1 (S220) acts in parallel to AMPK-FNIP1-TFEB mitochondrial biogenesis signaling to optimize mitochondrial fuel utilization, enhancing ATP production and exercise capacity. Thus, FNIP1 is a multifunctional AMPK effector for mitochondrial adaptation to exercise. These findings implicate a target for exercise tolerance in the health and disease of the skeletal muscle.

## RESULTS

### AMPK directly phosphorylates FNIP1 (S220) in vitro and in vivo

**Fig. 1.**
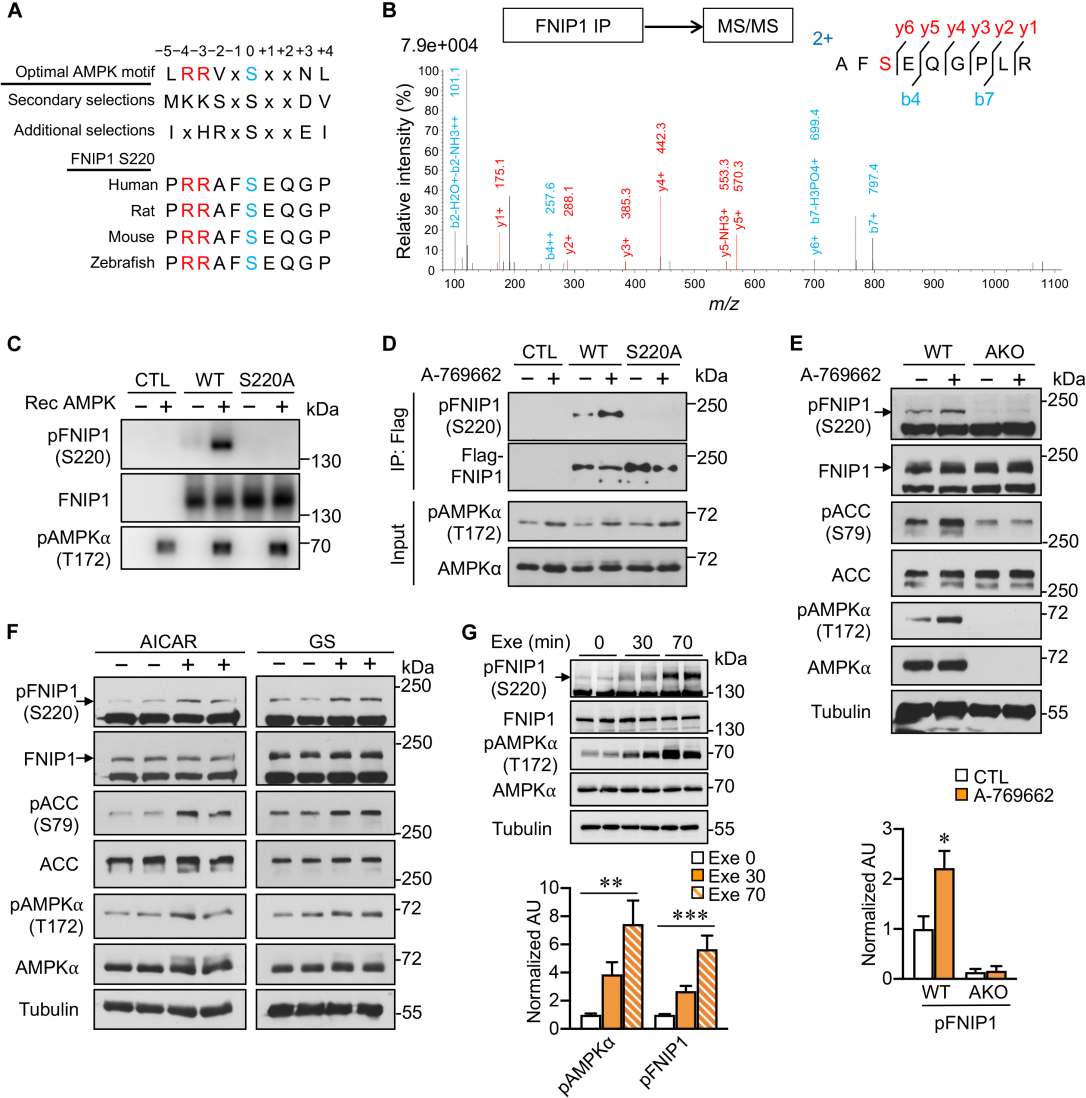
AMPK phosphorylates FNIP1 (S220) in vitro and in vivo. (**A**) Clustal alignment of AMPK substrate consensus sequences and the conserved S220 phosphorylation site on FNIP1. (**B**) FNIP1 phosphorylation peptide identification by MS analysis. Flag-FNIP1 was immunoprecipitated (IP), and the phosphopeptide containing phosphorylated S220 was identified by liquid chromatography–MS/MS. *m*/*z*, mass/charge ratio. (**C**) AMPK in vitro kinase assays with FNIP1. Flag-FNIP1 (WT) or (S220A) IPs from compound C-pretreated HEK293T cells were used as substrates for recombinant (Rec) AMPK; phosphorylation was detected by the FNIP1 (S220) antibody. CTL, control. (**D**) FNIP1 (S220) phosphorylation in response to AMPK activation in HEK293T cells. HEK293T cells transfected with Flag-FNIP1 (WT) or (S220A) were treated with vehicle or 100 μM A-769662 for 1 hour. (**E** and **F**) AMPK-mediated FNIP1 (S220) phosphorylation in primary myotubes. (E) Top: Primary myotubes from WT or AMPKα1/α2 double knockout (AKO) muscles were treated with either vehicle or 300 μM A-769662 for 1 hour and immunoblotted with the indicated antibodies. Bottom: Quantification of FNIP1 (S220) phosphorylation by pFNIP1/FNIP1 signal ratios. AU, arbitrary units. (F) Primary myotubes were treated with AICAR (2 mM; for 1 hour) or glucose starvation (GS) for 2 hours. Lysates were immunoblotted with the indicated antibodies. (**G**) Top: Representative immunoblots with white vastus lateralis (WV) muscle total protein extracted from acutely exercised (Exe) WT FNIP1 Tg mice with the indicated antibodies. Bottom: Quantification of FNIP1 (S220) and AMPKα (T172) phosphorylation by pAMPK/AMPK and pFNIP1/FNIP1 signal ratios. *n* = 4 mice per group. Error bars are shown as the means ± SEM. Experiments (C) to (F) were repeated at least three times. *P < 0.05; **P < 0.01; ***P < 0.001. The *P* value was determined by one-way analysis of variance (ANOVA) coupled to Fisher’s least significant difference (LSD) post hoc test.

AMPK and its effector phosphorylation are essential for mitochondrial regulation under energetic stress conditions, but the full repertoire of AMPK biological targets related to mitochondria in the skeletal muscle in response to exercise remains unclear. Previous studies reported that FNIP1, a key mitochondrial regulator, preferentially binds to phosphorylated AMPK and regulates AMPK activity (*25*). However, we observed a comparable FNIP1 binding capability with total AMPK or phosphorylated AMPK upon treatment with the AMPK activator A-769662 (fig. S1A). We further tested whether FNIP1 is an AMPK phosphorylation effector, as it contains a highly conserved optimal AMPK phosphorylation consensus (Fig. 1A). FNIP1 immunoprecipitated from human embryonic kidney (HEK) 293T cell lysate was phosphorylated at the S220 residue, as identified by phosphopeptide mass spectrometry (MS) (Fig. 1B). Specific antibody was generated against pS220 of FNIP1 [pFNIP1 (S220)], which recognizes recombinant AMPK-phosphorylated wild-type (WT) FNIP1 but not the nonphosphorylatable mutant FNIP1 (S220A) in vitro (Fig. 1C). An increase in pFNIP1 (S220) signals was induced with FNIP1 (WT) but not FNIP1 (S220A) in HEK293T cells treated with A-769662 (Fig. 1D). These data validated the effectiveness of the pFNIP1 (S220) antibody and the authentication of FNIP1 (S220) phosphorylation by AMPK. Furthermore, the A-769662–induced endogenous FNIP1 (S220) phosphorylation requires AMPK, as it was abolished in mouse primary myotubes isolated from AMPKα1/α2 double knockout mice (Fig. 1E) (*31*), and this phosphorylation could also be stimulated in myocytes by various AMPK activators, such as 5-Aminoimidazole-4-carboxamide-1-beta-4-ribofuranoside (AICAR), glucose starvation, and A-769662 (Fig. 1F and fig. S1, B and C). S220 phosphorylation was increased with AMPK phosphorylation stimulated by exercise training in muscle tissue from muscle-specific WT FNIP1–overexpressing (WT FNIP1 Tg) mice (Fig. 1G), implying that this phosphorylation is functionally important for the AMPK-mediated physiological response to exercise. We further tested whether the phosphorylation affected the AMPK-FNIP1 interaction; although S220-phosphorylated FNIP1 was structurally different from WT as predicted by AlphaFold2 and SWISS-MODEL modeling (fig. S1, D and E), both FNIP1 (S220A) and FNIP1 (S220D) exhibited no difference in AMPK binding compared to FNIP1 (WT) (fig. S1F). FNIP1/FLCN complex functions as a GAP for RagC and RagD GTPases and acts as an amino acid sensor for amino acid–induced mTORC1 activation (*28**,* *29*); however, *Fnip1* knockdown by small interfering RNA (siRNA) in HEK293T cells did not affect amino acid–induced S6K phosphorylation, a canonical indicator of mTORC1 activity (fig. S1G). Neither FNIP1 (S220A) nor FNIP1 (S220D) rescue in the FNIP1-deficient cells had any effect on S6K phosphorylation induced either by amino acid or glucose (fig. S1, G and H), suggesting that FNIP1 (S220) phosphorylation by AMPK does not affect nutrient-induced mTORC1 activity. Together, these data strongly suggest that FNIP1 is a constitutive AMPK binding partner and a bona fide phosphorylation substrate.

### Skeletal muscle–specific deletion of FNIP1 leads to enhanced exercise capacity

**Fig. 2.**
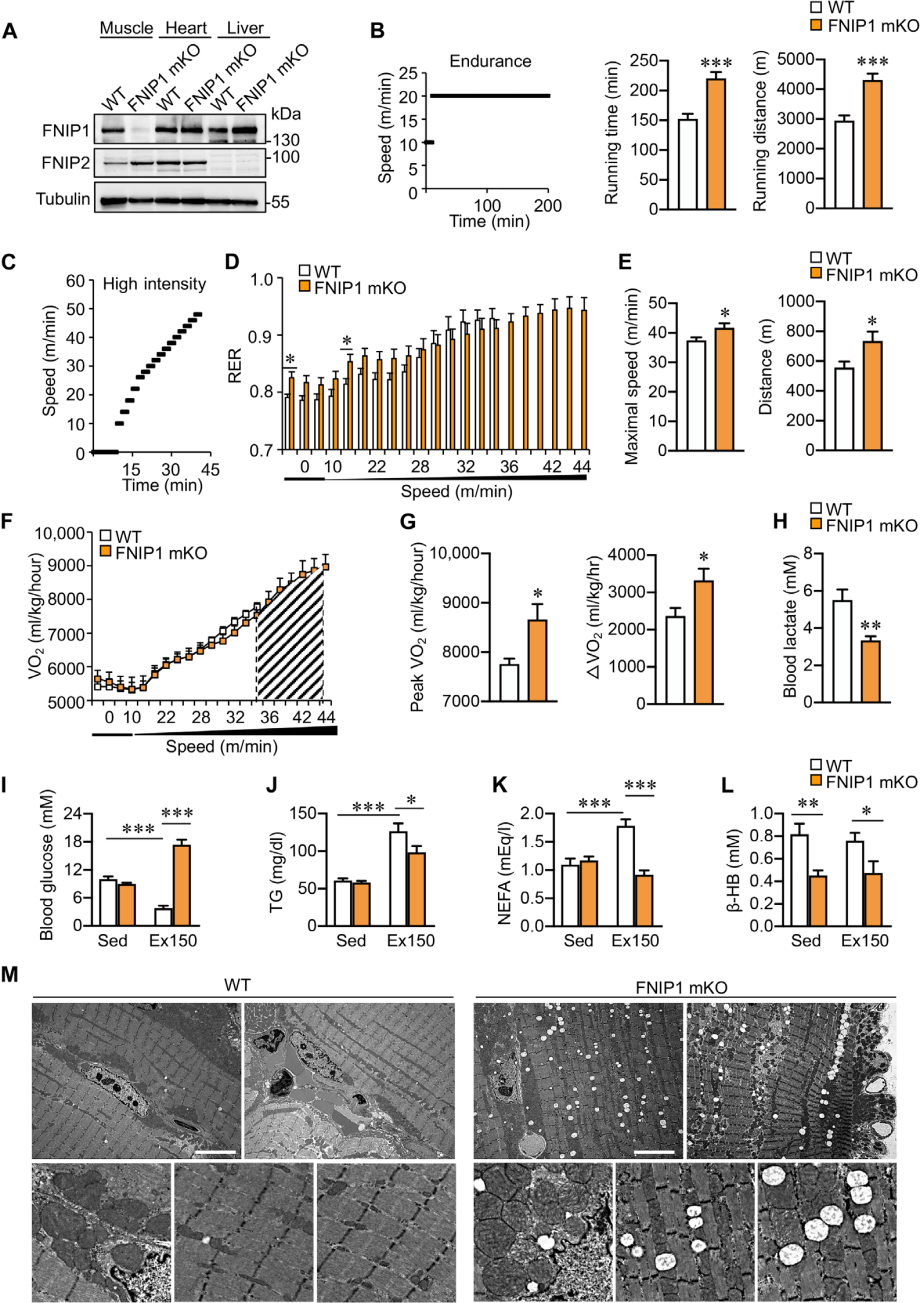
Deletion of muscle FNIP1 enhances exercise capacity in mice. (**A**) Representative immunoblotting analysis of FNIP1 and FNIP2 expression in tissue from indicated mice. *n* = 4 to 5 mice per group. (**B**) Exercise endurance test in FNIP1 mKO mice. Left: A schematic depicting increments of speed over time. Middle and right: Bars represent the running time and distance for 8-week-old male mice on a motorized treadmill. *n* = 11 mice per group. (**C** and **D**) Exercise capacity test in FNIP1 mKO mice. (C) The schematic depicts increments of speed over time. (D) The RER in FNIP1 mKO mice. *n* = 7 mice per group. (**E**) Maximal speed and total running distance in FNIP1 mKO mice. *n* = 7 mice per group. (**F**) VO_2_ (oxygen consumption) during exercise. The gray-shaded area under the VO_2_ line illustrates the difference in speed to exhaustion in FNIP1 mKO mice compared to WT controls. *n* = 7 mice per group. (**G**) Peak VO_2_ during exercise in FNIP1 mKO mice. *n* = 6 to 7 mice per group. (**H**) Blood lactate levels in indicated mice after a 25-min exercise. *n* = 11 mice per group. (**I** to **L**) Blood glucose (I), TG (J), NEFA (K), and β-hydroxybutyrate (β-HB) (L) levels were determined in indicated mice at rest (Sed) or after 150-min exercise (Ex150). *n* = 4 to 5 mice per group. (**M**) Representative electron micrographs of soleus muscle showing mitochondria in sections from indicated mice. *n* = 3 mice per group. Scale bars, 5.0 μm. Error bars are shown as the means ± SEM. *P < 0.05; **P < 0.01; ***P < 0.001. The *P* value was determined by Student’s *t* test [(B), (D), (E), (G), and (H)] or one-way ANOVA coupled to Fisher’s LSD post hoc test [(I) to (L)].

Despite FNIP1’s emerging role in the regulation of mitochondrial and muscle fiber–type programming (*31**,* *33*), it remains unclear whether and how FNIP1 contributes to the metabolic adaptation and plasticity of skeletal muscle during physical exercise. To address this, we crossed *Fnip1*-floxed mice (*Fnip1*^f/f^, exon 6 flanked by two loxP sites) with human skeletal actin Cre mice to create skeletal muscle–specific FNIP1-knockout (FNIP1 mKO) mice. FNIP1 protein and mRNA expression was markedly reduced in muscle, but not in other tissues, in FNIP1 mKO mice compared to WT littermates (Fig. 2A and fig. S2, A and B), while the protein expression of FNIP2, a close homolog of FNIP1, was increased in FNIP1 mKO muscle relative to WT littermates (Fig. 2A and fig. S2C). To determine the physiological impact of FNIP1 deficiency in muscle, we assessed the acute running endurance performance of the FNIP1 mKO mice using a run-to-exhaustion protocol on a motorized treadmill. Notably, FNIP1 mKO mice could run for longer times and distances (~40%) than their WT littermates (Fig. 2B). A forced maximal exercise capacity test revealed that FNIP1 mKO mice ran longer distances and achieved higher maximal speeds than WT controls, despite a similar energy substrate utilization indicated by the respiratory exchange ratio (RER) during the course of exercise (Fig. 2, C to E). Consistent with the enhanced exercise capacity, FNIP1 mKO mice consumed more oxygen (as reflected by peak VO_2_) with higher maximal peak oxygen consumption (VO_2_ max) than WT controls during the exercise period (Fig. 2, F and G); postexercise blood lactate levels, an indicator of glycolysis, were decreased in FNIP1 mKO mice (Fig. 2H); higher blood glucose, lower blood triglyceride (TG), and nonesterified fatty acid (NEFA) levels were observed after 150 min of endurance exercise in FNIP1 mKO mice (Fig. 2, I to K). Consistently, blood ketone body β-hydroxybutyrate levels were decreased in FNIP1 mKO mice under both basal and postexercise conditions (Fig. 2L), suggesting an enhancement of mitochondrial metabolism and exercise capacity. Such an “athletic” phenotype was reflected by the ultrastructure change of mitochondria in the muscle, as we observed by electron microscopy. The mitochondrial area was prominently increased in the FNIP1 mKO muscles compared to WT control muscles. Moreover, the amount of intramyocellular lipid droplets increased, indicating an increased reliance on lipids as energy substrates during running in FNIP1 mKO muscles (Fig. 2M). Together, these results demonstrated a change in metabolic flexibility with enhanced mitochondrial activity and exercise endurance in FNIP1 mKO mice.

### Exercise-induced FNIP1 (S220) phosphorylation controls mitochondrial metabolism and exercise performance in mice

**Fig. 3.**
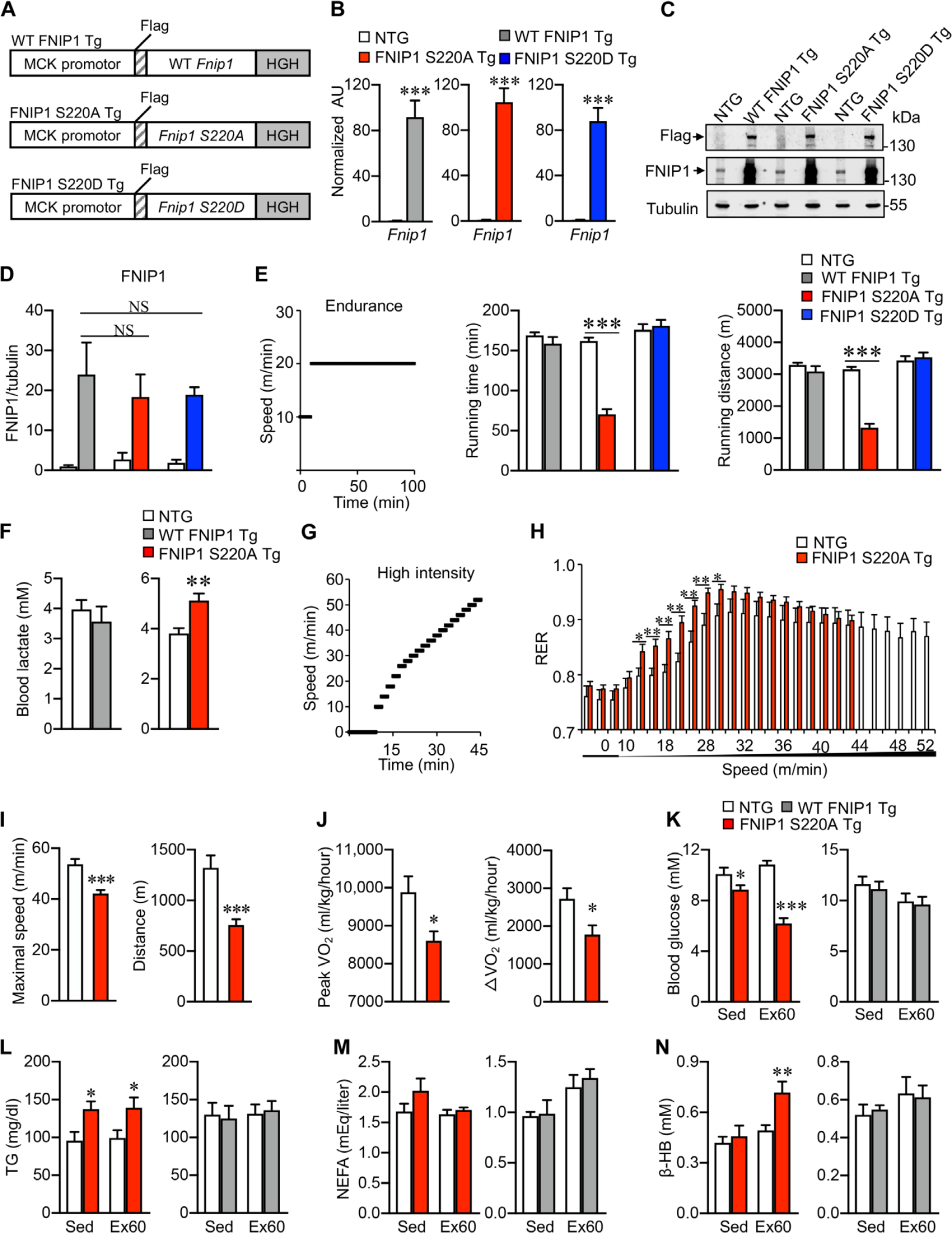
FNIP1 (S220) phosphorylation by AMPK regulates mitochondrial function and exercise performance in mice. (**A**) Schematic diagram depicting the *Mck*-driven WT *Fnip1*, S220A, and S220D transgenes. HGH, human growth hormone. (**B**) Quantitative reverse transcription polymerase chain reaction (RT-PCR) analysis of mRNA levels of *Fnip1* transgenes from the gastrocnemius (GC) muscle of the indicated transgenic (Tg) mice. *n* = 5 to 12 mice per group. (**C**) Endogenous and Tg FNIP1 expression in muscle tissues. Representative immunoblotting analysis of protein extracts from the WV muscles of the indicated Tg mice with the indicated antibodies. *n* = 3 mice per group. (**D**) Quantification of FNIP1 by signal ratios normalized to tubulin. (**E**) Exercise endurance test in *Fnip1* Tg mice. Left: A schematic depicting increments of speed over time. Middle and right: Bars represent the mean running time and distance for the indicated Tg mice on a motorized treadmill. *n* = 6 to 13 mice per group. (**F**) Blood lactate levels in WT FNIP1 Tg, S220A Tg, and NTG controls. *n* = 3 to 13 mice per group. (**G** to **J**) Exercise capacity test for *Fnip1* Tg mice. *n* = 6 to 10 mice per group. The schematic depicts the increments of speed over time (G). The RER during a graded exercise regimen (H), Maximal speed and total distance (I) as well as peak VO_2_ and peak ΔVO_2_ during exercise (J). (**K** to **N**) Blood glucose (K), TG (L), NEFA (M), and β-HB (N) levels at rest (Sed) or after 60 min of exercise (Ex60). *n* = 4 to 9 mice per group. Error bars are shown as the means ± SEM. *P < 0.05; **P < 0.01; ***P < 0.001. NS, not significant. The *P* value was determined by Student’s *t* test [(B), (E), (F), and (H) to (J)] or one-way ANOVA coupled to Fisher’s LSD post hoc test [(D) and (K) to (N)].

To pinpoint the potential function of FNIP1 (S220) phosphorylation by AMPK in mitochondrial regulation in the skeletal muscle, muscle-specific transgenic (Tg) mice with FNIP1 (S220A) and FNIP1 (S220D) expressed under the control of the muscle creatine kinase promoter (hereafter indicated as S220A Tg and S220D Tg) were established using the same strategy as that of WT FNIP1 Tg lines (Fig. 3A) (*31*). These three *Fnip1* Tg lines (WT FNIP1 Tg, S220A Tg, and S220D Tg) exhibited similar *Fnip1* mRNA levels in muscle tissues with 80- to 120-fold changes compared to the nontransgenic (NTG) littermate control mice (Fig. 3B). Consistently, immunoblotting experiments confirmed that the Flag-FNIP1 protein was overexpressed at equivalent levels in the muscles of all three mouse lines (Fig. 3, C and D). To examine the relevance of FNIP1 (S220) phosphorylation in exercise physiology, we performed treadmill-running tests comparing WT FNIP1 Tg, S220A Tg, S220D Tg, and NTG littermates. S220A Tg mice displayed a reduction in running time and distance, while there were no differences for S220D Tg or WT FNIP1 Tg relative to NTG controls (Fig. 3E). Consistent with reduced exercise tolerance, S220A Tg mice, but not WT FNIP1 Tg mice, showed higher levels (~35%) of blood lactate after exercise, indicating increased glycolysis (Fig. 3F). To further evaluate muscle fuel utilization during exercise, NTG or S220A Tg mice were also subjected to a forced maximal exercise capacity test (VO_2max_ test) consisting of increasing speed every 2 min until exhaustion. S220A Tg mice had a higher RER than NTG controls during exercise, and this was accompanied by reduced exercise tolerance (Fig. 3, G and H). In addition, upon high-intensity exercise challenge, S220A Tg mice showed decreased maximal speed and distance compared to their NTG control littermates (Fig. 3I). This is consistent with observations that S220A Tg mice consumed less oxygen during the exercise period (as reflected by peak ΔVO_2_) than NTG controls (Fig. 3J). Furthermore, S220A Tg mice showed lower blood glucose levels than NTG controls under basal conditions and after 60 min of endurance exercise (Fig. 3K), while blood TG was higher under basal conditions and after exercise in S220A Tg mice (Fig. 3L). However, there were no changes in blood glucose and TG levels in WT FNIP1 Tg mice relative to NTG controls under basal and postexercise conditions (Fig. 3, K and L). There were no changes in fatty acid levels in either WT FNIP1 Tg mice or S220A Tg mice relative to NTG controls (Fig. 3M). Notably, blood ketone body β-hydroxybutyrate levels, mirroring the oxidation of blood fatty acids, also increased in S220A Tg but not in WT FNIP1 Tg mice after exercise (Fig. 3N). Muscle glycogen is well known as a key energy source for contraction and is crucial for sustaining prolonged exercise. We found that glycogen content in the muscle of FNIP1 mKO mice was higher than that of WT mice, while it decreased in S220A Tg mice and was not changed in S220D Tg or WT FNIP1 Tg mice compared to their control littermates either by periodic acid–Schiff staining or glycogen content measurement (fig. S3, A and B), indicating that FNIP1 and its S220 phosphorylation by AMPK might contribute to the regulation of muscle glycogen metabolism. Together, these results demonstrate that FNIP1 (S220) phosphorylation by AMPK links metabolic flexibility to exercise performance through the promotion of mitochondrial function.

### FNIP1 (S220) phosphorylation controls mitochondrial ETC complex formation without affecting mTORC1-TFEB signaling

**Fig. 4.**
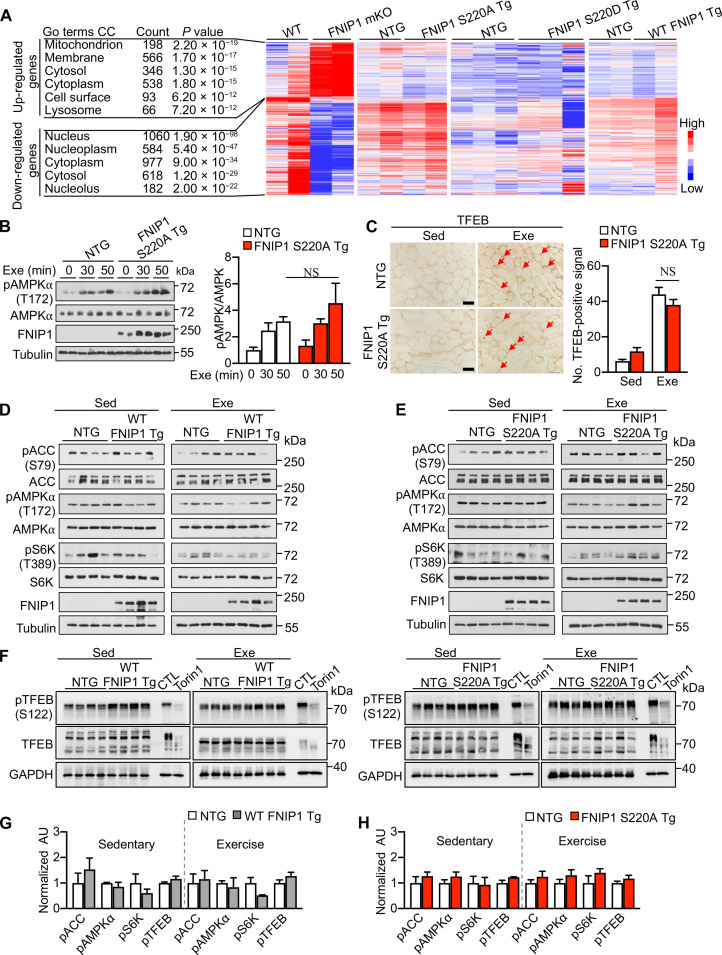
FNIP1 (S220) phosphorylation regulates muscle mitochondrial function without affecting TFEB signaling. (**A**) Gene expression profiling of GC muscle in mice. Heatmap analysis of DEGs in muscles of WT, FNIP1 mKO, and different transgenic (Tg) mice compared with NTG controls (*n* = 2 to 3 biological samples per group). DEGs between different groups were analyzed by Gene Ontology (GO), and mRNA fold change is indicated by colored bar. (**B**) Left: Representative immunoblots of muscle protein extracts from indicated mice following acute running exercise (Exe). Right: Quantification of AMPKα (T172) phosphorylation by pAMPK/AMPK signal ratios. *n* = 4 mice per group. (**C**) Left: Representative cross-sectional images of plantaris muscle stained with anti-TFEB antibody from indicated mice with or without exercise (Sed or Exe). Scale bars, 50 μm. Right: Quantification of TFEB-positive nuclear localization. *n* = 3 mice per group. (**D** and **E**) mTORC1 and AMPK signaling in *Fnip1* Tg muscle. Immunoblotting analysis of WV muscle lysates from sedentary (Sed) and 60-min exercised (Exe) mice of indicated genotype. *n* = 4 mice per group. (**F**) TFEB phosphorylation analysis in *Fnip1* Tg muscle. Immunoblotting analysis of muscle lysates from sedentary and 60-min exercised mice of indicated genotype. To monitor the band shift of TFEB, lysates of HEK293T cells with or without Torin1 treatment (CTL) were loaded as controls. (**G** to **H**) Quantification of pACC (S79), pAMPKα (T172), pS6K (T389), and pTFEB (S122), phosphorylation by signal ratios normalized to NTG controls (=1.0) during sedentary or exercise conditions from (D) to (F). *n* = 4 to 8 mice per group. Error bars are shown as the means ± SEM. NS, *P* > 0.05 was determined by one-way ANOVA coupled to Fisher’s LSD post hoc test [(B) and (C)]. No statistical significance in (G) and (H) calculated by Student’s *t* test.

**Fig. 5.**
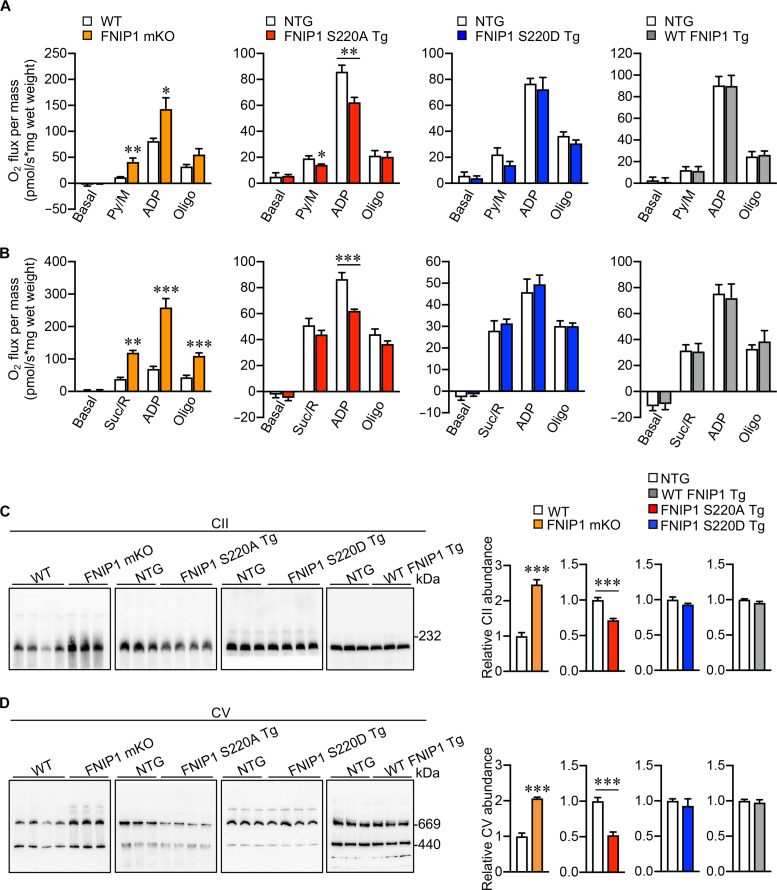
FNIP1 (S220) phosphorylation regulates mitochondrial ETC complex formation and respiration capability in muscle. (**A** and **B**) Mitochondrial respiration rate analysis in FNIP1 mKO and transgenic (Tg) muscle tissues. Mitochondrial respiration rates were determined from the extensor digital longus (EDL) muscle of the indicated genotypes using pyruvate (A) or succinate (B) as substrates. Pyruvate/malate (Py/M)– or succinate/rotenone (Suc/Rot)–stimulated, adenosine diphosphate (ADP)–dependent respiration, and oligomycin-induced (Oligo) respiration are shown. *n* = 4 to 7 mice per group. (**C** and **D**) The effects of FNIP1 (S220) phosphorylation on mitochondrial ETC complex assembly in the muscle. Mitochondrial proteins were extracted from the indicated mice after exercise and analyzed by BN-PAGE as described in Materials and Methods. Western blotting analysis was performed with anti-SDHA (CII) (C) and anti-ATP5A (CV) (D) antibodies. Equal total mitochondrial proteins were loaded, and the band gray values were quantified using ImageJ. WT and FNIP1 mKO mice, exercise for 150 min; NTG and *Fnip1* Tg mice, exercise for 60 min. *n* = 3 to 8 mice per group. Error bars are shown as the means ± SEM. *P < 0.05; **P < 0.01; ***P < 0.001. The *P* value was determined by Student’s *t* test.

To delineate how FNIP1 (S220) phosphorylation regulates mitochondrial function by exercise-induced AMPK activation in muscle tissue, we performed transcriptome analysis by whole-genome gene expression profiling in the gastrocnemius (GC) muscle from FNIP1 mKO and WT control mice, S220A Tg, S220D Tg, WT FNIP1 Tg, and NTG control mice, respectively. A total of 3550 differentially expressed genes (DEGs) were detected in FNIP1 mKO muscle compared to WT controls (Fig. 4A and fig. S3, C and D), including mitochondrial or lysosomal biogenesis signature genes related to PGC1α and TFEB (Fig. 4A); however, no such DEGs were detected in S220A Tg, S220D Tg, and WT FNIP1 Tg muscle relative to NTG controls (Fig. 4A and fig. S4A). This result suggested that FNIP1 (S220) phosphorylation by AMPK did not affect TFEB-related gene expression in muscles as the whole FNIP1 knockout. A recent study reported that mTORC1-TFEB-PGC1α signaling is important for AMPK-FNIP1–mediated mitochondrial biogenesis (*18*). To clarify if FNIP1 (S220) phosphorylation was involved in this process, we tested AMPK and mTORC1-TFEB signaling in S220A Tg muscle tissues. The AMPK activity was comparable in S220A Tg and NTG muscle either in sedentary or in exercised conditions (Fig. 4B); FNIP1 (S220A Tg) muscle exhibited normal mTORC1 activity toward canonical substrates S6K; nuclear translocation of TFEB, a noncanonical mTORC1 substrate (*40*), was also not changed between S220A Tg and NTG muscle tissues by TFEB histochemical staining at both baseline and exercise conditions (Fig. 4, C to H). Neither phospho-TFEB (S122) nor total TFEB band shift (indicative of TFEB overall phosphorylation levels) were changed in both WT FNIP1 Tg and S220A Tg muscle at baseline or exercise conditions compared to the NTG control (Fig. 4, F to H). These data collectively suggest that FNIP1 (S220) phosphorylation is likely not directly involved in the regulation of mTORC1-TFEB–mediated gene expression in the skeletal muscle. Nevertheless, the regulatory effects of FNIP1 (S220) phosphorylation on mitochondria in the muscle were prominent, as both pyruvate- and succinate-driven mitochondrial respiration rates were markedly induced in the muscle of FNIP1 mKO mice compared to WT controls. On the contrary, FNIP1 (S220A) suppressed mitochondrial respiration rates, while there was no difference in FNIP1 (S220D Tg) or WT FNIP1 Tg muscle (Fig. 5, A and B), indicating that S220 phosphorylation is required for normal mitochondrial respiration rates.

Recent studies have revealed that AMPK can localize to mitochondria in the skeletal muscle (*41*). To pinpoint the potential mechanism, we checked the subcellular location of FNIP1. Immunofluorescence costaining of Flag-FNIP1 with the inner mitochondrial membrane marker protein succinate dehydrogenase complex flavoprotein subunit A (SDHA) revealed overlapping signals (fig. S4B). Moreover, we isolated mitochondria from adult mouse skeletal muscle by sucrose gradient fractionation and performed immunoblotting using anti-AMPK and anti-FNIP1 antibodies. As shown in fig. S4C, we readily detected both FNIP1 and AMPK in these enriched mitochondrial fractions (fig. S4C). Notably, we did not detect FLCN in these enriched mitochondrial fractions (fig. S4C), implicating that AMPK/FNIP1, but not the GAP catalytic protein FLCN, is subcellularly localized or associated with mitochondria in the skeletal muscle. We also took advantage of the skeletal muscle of FNIP1 mKO mice for fractionation, and we observed a complete loss of FNIP1 but not AMPK in enriched mitochondrial fractions (fig. S4D); again, we could not detect FLCN in the mitochondrial fractions (fig. S4D). Using muscle-specific FNIP1 (WT) and FNIP1 (S220A Tg) muscle tissues, we identified approximately 600 potential FNIP1 (WT)– or FNIP1 (S220A)–binding proteins in exercising muscle tissue of the corresponding mice by the coimmunoprecipitation (CoIP)–MS method (fig. S4E). Proteomic analysis showed that both FNIP1 (WT) and FNIP1 (S220A) interacted with major mitochondrial proteins, such as the respiratory complex subunits (fig. S4F). Notably, FNIP1 (S220A) specifically interacted with additional mitochondrial subunits that were not observed for FNIP1 (WT), including proteins involved in reduced form of nicotinamide adenine dinucleotide (oxidized form) metabolism and sulfur cluster binding (fig. S4G). These data suggested that nonphosphorylated FNIP1 (S220A) could enhance its interaction with mitochondria. To further determine the effects of FNIP1 phosphorylation on mitochondria, we checked the mitochondrial ETC (electron transport chain) complex formation during exercise. Blue-native polyacrylamide gel electrophoresis (BN-PAGE) of digitonin-treated mitochondria followed by immunoblotting with specific mitochondrial subunit antibodies revealed that knockout of FNIP1 increased mitochondrial ETC complex formation during exercise. In contrast, S220A suppressed mitochondrial complex II (CII) and complex V (CV) formation, and there were no such effects in S220D Tg or WT FNIP1 Tg muscle during exercise (Fig. 5, C and D). It has been reported that mitochondrial chaperone genes regulate mitochondrial function by modulating ETC complex assembly (*42**,* *43*). We also sought to determine whether mitochondrial chaperone gene expression is affected in *Fnip1* Tg muscles. However, we did not detect mRNA level changes for most mitochondrial chaperones except a mild decrease in *Clpp* mRNA in WT FNIP1 Tg and *Dnaja3* mRNA in S220D Tg muscles compared to NTG controls (fig. S5, A to C). We also found that *Dnaja3* and *Foxred1* mRNA levels were mildly decreased in S220A Tg muscle (fig. S5B). We found that *Ppargc1*α mRNA levels were mildly decreased in S220A Tg muscle (fig. S5B), whereas *Ppargc1*α gene expression was not changed in WT FNIP1 Tg or S220D Tg muscles (fig. S5, A and C). Collectively, these results suggest that FNIP1 (S220) phosphorylation contributes to mitochondrial ETC complex formation rather than affecting TFEB signaling in the skeletal muscle during exercise.

## DISCUSSION

AMPK is the metabolic fuel gauge that senses changes in the intracellular AMP/ATP ratio in response to physical exercise. Activation of skeletal muscle AMPK by exercise or exercise mimicking drugs enhances mitochondrial metabolism and has become a powerful therapeutic strategy for type 2 diabetes and associated metabolic disorders. AMPK controls multiple aspects of mitochondrial function by phosphorylating effector proteins in diverse metabolic pathways involved in oxidative respiration and fatty acid oxidation. The delineation of the full repertoire of AMPK targets and mechanisms involved in the beneficial effects of exercise on muscle mitochondrial metabolism has implications for therapeutic approaches for many human diseases, including metabolic disorders and muscular dystrophy.

In this study, we identified that FNIP1, a hypothetical mitochondrial gatekeeper (*44*), was phosphorylated at the S220 site, contributing to AMPK regulation of mitochondrial function in response to exercise in mouse models. We originally found that muscle-specific FNIP1 KO mice develop many features of an exercise-trained athletic phenotype, including increased endurance, enhanced mitochondrial capacity, and fat burning. While muscle physiological exercise capacity is normal and mitochondrial function is unchanged in muscle-specific WT FNIP1 Tg and S220D Tg transgenic mice, the lack of FNIP1 (S220) phosphorylation (S220A Tg) leads to compromised mitochondrial function and an exercise intolerance phenotype in mice. These results highlighted that single-site phosphorylation at S220 of FNIP1 by exercise-activated AMPK contributes to mitochondrial regulation and muscle exercise physiology.

The regulation of mitochondrial metabolism by AMPK in exercising muscle occurs at multiple levels, including de novo mitochondrial biogenesis and mitochondrial proteomic remodeling (*8**,* *17**,* *23*). Previous studies have demonstrated that the TFEB and PGC1α transcriptional regulatory circuit, including the nuclear receptors PPARs and ERRs, are key transducers of exercise-responsive mitochondrial gene expression for mitochondrial biogenesis and fuel metabolism (*12**,* *20**,* *45**,* *46*)*.* Evidence is also emerging that mitochondrial proteome remodeling induced by exercise in skeletal muscle can affect mitochondrial function and muscle performance (*22**,* *23*). Our results suggested that exercise-induced FNIP1 (S220) phosphorylation by AMPK participated in the regulation of mitochondrial function and related muscle fuel catabolism, possibly through its compartmentalized subpool on mitochondria. The discovery of a mitochondrial pool of FNIP1 and its importance for mitochondrial respiration control underscores the complexity of energetic monitoring in the skeletal muscle. These data imply a mechanism for how AMPK regulates mitochondrial function through its constitutive binding partner FNIP1 in the exercising muscle. S220 is not conserved in FNIP2, a close homolog of FNIP1, which suggests that FNIP1 likely has a unique AMPK phosphorylation–dependent regulatory mechanism that FNIP2 lacks.

An interesting finding of our study is that FNIP1 (S220) phosphorylation by AMPK participates in the regulation of mitochondrial function without affecting muscle TFEB signaling. Our results suggested that FNIP1 (S220) phosphorylation acts independently of TFEB signaling to regulate mitochondrial ETC complex assembly and fuel metabolism. First, RNA sequencing (RNA-seq) data revealed no expression change of TFEB target genes in S220A Tg, S220D Tg, or WT FNIP1 Tg muscle compared to NTG controls, despite prominent lysosomal or mitochondrial genes being detected in FNIP1 mKO muscle compared to WT controls; second, we found that FNIP1 deficiency, as previously reported, did not affect mTORC1 activity toward its canonical substrate S6K phosphorylation, and rescue of both FNIP1 (S220A) and FNIP1 (S220D) phosphomutants exhibited no effects on S6K phosphorylation in cells or in muscle tissues; third, nuclear translocation of TFEB, a noncanonical mTORC1 substrate, was also not changed either in sedentary or in exercised muscle; neither phospho-TFEB (S122) nor total TFEB band shift (indicative of TFEB overall phosphorylation levels) were changed in both WT FNIP1 Tg and S220A Tg muscle at baseline or exercise conditions compared to the NTG control; fourth, there exists a FNIP1 subpool on mitochondria, and FNIP1 (S220A) showed a differential interactive network from FNIP1 (WT) for mitochondrial proteins. Mitochondrial ETC complex formation and respiratory function were compromised in the S220A Tg muscle during exercise. Mitochondrial ETC formation in the S220A Tg muscle is likely not driven by the mild changes in chaperones for ETC complexes, although a reduced muscle *Ppargc1*α may contributes to impaired mitochondrial function in the S220A Tg muscle. Our results provide evidence that phosphorylation of FNIP1 (S220) by AMPK controls exercise capacity and regulates mitochondrial ETC function. The precise mechanisms involved in the regulation of mitochondrial ETC formation via FNIP1 (S220) need further dissection based on these discoveries. While we cannot exclude the possibility that FNIP1 S220A acts indirectly on mitochondrial ETC formation (via some indirect interaction with key ETC formation regulatory proteins or even potentially via indirect regulation at the transcriptional level), our findings suggest that there exists an AMPK-FNIP1 subpool on mitochondria that may be involved in the regulation of mitochondrial ETC assembly, possibly in an FLCN-independent manner as FLCN, the catalytic subunit of GAP activity toward RagC/D, was not present as a mitochondrial protein. Future studies will be necessary to further delineate the mitochondrial-localized AMPK-FNIP1 mechanism in regulating muscle physiology. It will also be of interest to investigate whether phosphorylation of S220 regulates the protein stability of FNIP1.

When this manuscript was being prepared, the Shaw group (*18*) had recently published a study that identified five AMPK phosphorylation sites, including S220 in FNIP1. By delicate cellular mechanistic analysis, they elegantly demonstrated that FNIP1 phosphorylation at these sites by AMPK was coordinated with RagC and mTORC1 to induce nuclear translocation of TFEB and subsequent PGC1α and ERRα transcription, thus contributing to mitochondrial biogenesis in response to mitochondrial poisoning. This and other previous studies have highlighted the important role of FNIP1 in TFEB signaling (*18**,* *19**,* *28**,* *37**,* *39**,* *47*). In our experimental system, no differences in TFEB (S122) phosphorylation, total TFEB band shift, or translocation were observed in WT FNIP1 Tg or S220A Tg muscle tissue. Our data here suggested that FNIP1 (S220) phosphorylation by AMPK is not essential or not sufficient for the inhibition of TFEB phosphorylation by mTORC1. Nevertheless, we cannot exclude the possibility that FNIP1 phosphorylation at additional site(s) by AMPK affects TFEB phosphorylation. Future studies aimed at dissecting the essential phosphorylation site(s) for TFEB regulation will likely require more stringent mouse models, including rescue of the FNIP1 mKO muscle with single-site mutants, such as FNIP1 (S220A) and FNIP1 (S220D), and with multiple-site mutants, such as FNIP1 (SA4) or FNIP1 (SA5), identified by Malik *et al.* (*18*).

Here, using genetic mouse models, we provided physiological evidence that single-site phosphorylation at S220 of FNIP1 by exercise-activated AMPK contributes to mitochondrial regulation through mitochondrial proteomic remodeling and ETC complex assembly in the skeletal muscle. Such a mechanism is in parallel to the AMPK-FNIP1-TFEB mitochondrial biogenesis mediated by multiple-site FNIP1 phosphorylation. Nevertheless, it is also plausible that both proteome or ETC complex remodeling and the biogenesis of mitochondria are coordinately regulated by AMPK phosphorylation of FNIP1.

## MATERIALS AND METHODS

### Experimental design

The objective of this study was to investigate the key effector(s) of AMPK and the regulation of muscle mitochondrial function and exercise performance. Using a combination of genetic mouse models and biochemical approaches, our study highlights the critical role of FNIP1 (S220) phosphorylation by AMPK in controlling mitochondrial function and muscle fuel utilization during exercise. These findings implicate a target for exercise tolerance in the health and disease of the skeletal muscle. The sample sizes are explicitly stated in the figure legends. No statistical methods were used to predetermine sample sizes. Sample sizes were determined on the basis of previous experiments using similar methodologies and are sufficient to account for any biological/technical variability. For all experiments, samples/animals were randomly allocated to experimental groups and processed. No data were excluded from the analyses. The investigators were not blinded to allocation during experiments and outcome assessment because the investigators needed to conduct genotyping polymerase chain reactions (PCRs) at the age of 2 weeks for the mice. Blinding was not relevant to the other experiments in cells because the investigators needed to know what cell type they had to culture and process the cells by themselves.

### Animal studies

All animal studies were conducted in strict accordance with the institutional guidelines for the humane treatment of animals and were approved by the Institutional Animal Care and Use Committee (IACUC) committees at the Model Animal Research Center of Nanjing University. The generation of *Fnip1* floxed mice has been described previously in detail (*48*). Briefly, the *Fnip1* targeting vector was generated by inserting two loxP sequences into intron 5 and intron 6. To generate mice with a muscle-specific disruption of the *Fnip1* allele, *Fnip1*^fl/fl^ mice were crossed with mice expressing Cre recombinase under the control of an human skeletal actin promoter (the Jackson laboratory, stock no. 006139) to achieve muscle-specific deletion of *Fnip1* (FNIP1 mKO). Deletion of exon 6 in the FNIP1 mKO mice resulted in a reading frameshift and premature termination codon in exon 7. The generation of FNIP1 transgenic (FNIP1 Tg) mice under the control of the muscle creatine kinase promoter (a kind gift from E. N. Olson, University of Texas Southwestern) has been described elsewhere (*31*), and the *Fnip1* transgene was expressed efficiently in a skeletal muscle–specific manner with no overexpression of FNIP1 protein in other tissues, such as the heart (*31*). Muscle-specific FNIP1 S220A/S220D Tg mouse models (FNIP1 S220A/S220D Tg) were established using the same strategy as that of WT FNIP1 Tg lines. Briefly, cDNA encoding the mouse *Fnip1* mutant gene was separately cloned and inserted into the EcoR V site downstream of the mouse *Mck* gene promoter. Site-directed mutagenesis was carried out using the QuikChange Kit (Stratagene) according to the manufacturer’s protocol: the amino acid S220 (S; 5′-TCT-3′), an FNIP1 phosphorylation site, was changed to Ala (A; 5′-GCT-3′) or Asp (D; 5′-GAT-3′), which mimicked dephosphorylation or phosphorylation, respectively. The transgene was linearized with Xho I and Sac II digestion and microinjected into C57BL/6J embryos by the Tg mouse facility at the Model Animal Research Center of Nanjing University. Tg mice were identified by PCR amplification of a 534–base pair product using primers specific for *Fnip1* (5′-TTTCCAACCTGCTTCATTCCACTCTTCA) and the human growth hormone polyadenylate component of the muscle creatine kinase (MCK) construct (5′-AAGATTGTGCCACTGCA). Male mice aged 8 to 12 weeks were used.

### Recombinant AMPK purification and in vitro kinase assay

AMPKα1, AMPKβ1, AMPKγ1, and Calcium/calmodulin-dependent protein kinase kinase β (CaMKKβ) were subcloned and inserted into the pET28b vector, and the bacterial expression and purification of recombinant AMPKα1β1γ1 and CaMKKβ were performed. Briefly, recombinant proteins were induced with 0.1 mM isopropyl-β-d-thiogalactopyranoside at 22°C overnight in *Escherichia coli* BL21(DE3) cells when the cell density reached 0.4 to 0.6 at 600 nm (optical density at 600 nm). Cells were pelleted and resuspended in lysis buffer containing 15% sucrose (w/v), 50 mM sodium phosphate (pH 7.5), 100 mM NaCl, 10 mM imidazole, and 1 mM β-mercaptoethanol with 1% Triton X-100. After sonication and centrifugation, the supernatant was collected and purified with Ni–nitrilotriacetic acid resin. After washing with lysis buffer containing 50 mM imidazole, the proteins were eluted with lysis buffer containing 250 mM imidazole and dialyzed overnight with dialysis buffer [50 mM tris-HCl (pH 7.5), 1 mM β-mercaptoethanol, and 1 mM EDTA] at 4°C to remove imidazole and then stored at −80°C until use (*49*).

For the in vitro kinase assay, 200 ng of recombinant AMPKα1β1γ1 complex was preincubated with 20 ng of CaMKKβ to be fully activated in reaction buffer [20 mM tris-HCl, 1 mM dithiothreitol, 5 mM MgCl_2_, and 8 nM ATP (pH 7.5)] in a 30°C water bath for 2 hours. Flag-FNIP1 (WT and S220A) was purified from HEK293T cells transfected with Flag-FNIP1 (WT and S220A). Cells were pretreated with the AMPK inhibitor compound C (10 μM) for 4 hours before harvest, which inhibited AMPK-dependent FNIP1 phosphorylation. Cell lysates were collected 36 hours after transfection, and Flag-FNIP1 was purified with Flag antibody. Protein A/G beads and 200 ng of eluted substrate Flag-FNIP1 (WT and S220A) protein were incubated with 20 ng of activated AMPK in reaction buffer in a 37°C water bath for 1 hour. After incubation, the reaction was terminated by SDS loading buffer and heated to 95°C for 10 min, followed by SDS-PAGE and immunoblotting with specific antibodies.

### MS analysis

To identify phosphorylation sites of FNIP1, pcDNA5-Flag-FNIP1 was expressed in HEK293T cells, and the cells were treated with A-769662 before harvest. For Flag-IP, cell lysates were incubated with anti-Flag M2 beads (Sigma-Aldrich, A2220) at 4°C overnight. IP of proteins and phosphorylated peptides/residues was performed by Shanghai Applied Protein Technology Co. Ltd. To identify FNIP1-interacting proteins, muscle tissues from FNIP1 Tg or FNIP1 S220A Tg mice were homogenized and then incubated with anti-Flag M2 beads (Sigma-Aldrich, A2220) at 4°C overnight. The samples were subjected to SDS-PAGE, and stained with Coomassie bright blue R-250. For MS identification, the gel portions containing the protein bands were excised, destained, dehydrated, and subjected to trypsin digestion. The resulting peptides were analyzed by a liquid chromatography–MS system (2D-NanoLC/TripleTOF5600) (*50*).

### Exercise stress test

Mice were acclimated (run for 9 min at 10 m/min, followed by 1 min at 20 m/min at a 10° incline) to the treadmill for two consecutive days prior to the experimental protocol. Low-intensity (endurance) exercise studies were conducted. Briefly, fed mice were run for 10 min at 10 m/min, followed by a constant speed of 20 m/min at a 10° incline until exhaustion. Tail blood was taken after exercise and measured for lactate (Lactate Scout, Senelab, Germany) according to the manufacturer’s instructions (*32*).

RERs during exercise were determined using a high-intensity exercise protocol. Briefly, mice were placed in an enclosed treadmill attached to the Comprehensive Laboratory Animal Monitoring System (Columbus Instruments) for 15 min at a 0° incline and 0 m/min. The mice were then challenged with 2-min intervals of increasing speed at a 0° incline. The increasing speeds used in the protocol were 10, 14, 18, 22, 26, 28, 30, 32, 34, 36, 38, 40, 42, 44, 46, 48, 50, and 52 m/min. Measurements were collected before the exercise challenge, throughout the challenge, and following failure.

### Blood and tissue chemistry

Blood glucose levels were determined using a OneTouch UltraMini glucose meter (OneTouch). Serum TG levels were determined using a TG Kit (Wako, 290-63701). Serum fatty acid levels were determined using a NEFA kit (Wako, 294-63601). Serum β-hydroxybutyrate levels were measured using the β-hydroxybutyrate (Ketone Body) Colorimetric Assay Kit (Cayman Chemical, 700190) according to the manufacturer’s instructions.

### Mitochondrial respiration studies

Mitochondrial respiration rates were measured in saponin-permeablized extensor digital longus muscle fibers with pyruvate or succinate as substrates. Briefly, the muscle fibers were separated and transferred to BIOPS buffer [7.23 mM K_2_EGTA, 2.77 mM CaK_2_EGTA, 20 mM imidazole, 20 mM taurine, 50 mM potassium 2-[*N*-morpholino]-ethanesulfonic acid, 0.5 mM dithiothreitol, 6.56 mM MgCl_2_, 5.7 mM ATP, and 14.3 mM phosphocreatine (pH 7.1)]. The muscle fiber bundles were then permeabilized with saponin (50 μg/ml) in BIOPS solution. Measurement of oxygen consumption in permeabilized muscle fibers was performed in buffer Z [105 mM potassium 2-[*N*-morpholino]-ethanesulfonic acid, 30 mM KCl, 10 mM KH_2_PO_4_, 5 mM MgCl_2_, bovine serum albumin (5 mg/ml), and 1 mM EGTA (pH 7.4)] at 37°C and in the oxygen concentration range of 220 to 150 nmol of O_2_/ml in the respiration chambers of an Oxygraph 2K (Oroboros Inc., Innsbruck, Austria). Following measurement of basal, pyruvate (10 mM)/malate (5 mM) or succinate (5 mM)/rotenone (10 μM) respiration, maximal [adenosine diphosphate (ADP)–stimulated] respiration was determined by exposing the mitochondria to 4 mM ADP. Uncoupled respiration was evaluated following addition of oligomycin (1 μg/ml). Respiration rates were determined and normalized to tissue wet weight using Datlab 5 software (Oroboros Inc., Innsbruck, Austria), and the data were expressed as “picomole of O_2_ per second per milligram wet weight” (*31*).

### Mitochondrial isolation and BN-PAGE

Mitochondria were isolated via sucrose gradient fractionation from fresh mouse muscle as previously described (*51*). Briefly, minced muscles from mice of different genotypes were homogenized with a glass dounce in fractionation (FRAC) buffer [10 mM Hepes-Na, 300 mM sucrose, and 0.2 mM EDTA (pH 7.2)] and then centrifuged twice at 800*g* for 20 min at 4°C, and the resulting supernatant was termed postnuclear lysate. The resulting supernatants were carefully transferred to a new tube and centrifuged at 8000*g* for 20 min at 4°C. The pellet was resuspended in FRAC buffer and spun again until a solid pellet formed at the bottom of the tube, designated the mitochondrial fraction. The whole-tissue lysates, postnuclear lysate, and mitochondrial fractions were resuspended in FRAC buffer and quantified by bicinchoninic acid (BCA) assay using Pierce BCA Assay Kit Protocol (Thermo Fisher Scientific).

BN-PAGE analysis was performed. A total of 250 μg of mitochondria isolated as described above were resuspended in solubilization buffer [50 mM NaCl, 50 mM imidazole, 2 mM 6-aminohexanoic, and 1 mM EDTA (pH 7.0)]. Then, the mitochondria were incubated with 20% digitonin on ice for 10 min. After centrifugation at 20,000*g* for 30 min at 4°C, the supernatants were collected. The samples were mixed with 50% glycerol and 5% Coomassie G-250 and subjected to 3.5 to 13% BN-PAGE for electrophoresis at 4°C. After the native gel electrophoresis was conducted at 100 V for 30 min, cathode buffer B (50 mM tricine, 7.5 mM imidazole, and 0.02% Coomassie brilliant blue G-250) was changed to cathode buffer B/10 (50 mM tricine, 7.5 mM imidazole, and 0.002% Coomassie brilliant blue G-250), and the running continued at 15 mA for ~3 hours. The gels were electroblotted on polyvinylidene difluoride membranes for immunoblotting (*51*). Equal amounts of total mitochondrial proteins were loaded, and the relative levels of protein were quantified by band gray values calculated with ImageJ software.

### Transmission electron microscopy

Mice were euthanized, and soleus muscles were dissected, cut into small pieces, and fixed in 2.5% glutaraldehyde in 0.1 M phosphate buffer overnight at 4°C. Then, the specimens were postfixed for 1.5 hours with 1% osmium tetroxide in phosphate buffer, dehydrated through a graded ethanol series, and embedded in Epon812. Ultrathin sections (70 nm) were prepared, stained with uranyl acetate and lead citrate, and examined by electron microscopy (JEOL-1200EX) at JiNan WeiYa Bio-Technology Co. Ltd. (Jinan, China) in a double-blind manner.

### Histologic analysis

Muscle tissue was frozen in isopentane that had been cooled in liquid nitrogen. Notably, the GC of each mouse was dissected as a whole. Ten-micrometer-thick serial GC muscle cross sections were cut from the knee cut side in a Leica CM1850 cryostat at −20°C and mounted on positively charged glass slides. Transverse sections collected from the widest part (mid-belly) of the GC muscle were used for histological comparison to maintain consistency between different mice.

For TFEB immunohistochemistry (IHC), GC muscle cross sections were washed three times with phosphate-buffered saline (PBS) for 5 min each, fixed in ice-cold 4% paraformaldehyde for 10 min, washed with PBS for 5 min, and then permeabilized with ice-cold 0.5% Triton X-100–PBS for 10 min. IHC was performed using the UltraSensitive SP (rabbit) IHC Kit (Maxim) according to the manufacturer’s instructions using rabbit TFEB antibodies (Bethyl Laboratories, A303-673A; 1:200). A 3,3′-Diaminobenzidine (DAB) staining kit (Maxim, DAB-0031) was used according to the manufacturer’s instructions for development.

Periodic acid–Schiff staining was used to detect glycogen accumulation. The muscle fibers were oxidized in 0.5% periodic acid for 5 min, rinsed three times in distilled water, and treated with Schiff’s reagent for 20 min. After extensive washing, the slides were counterstained with hematoxylin and lastly sealed with neutral resin.

### Glycogen extraction and content measurement

For measurement of muscle glycogen, muscle tissue was acid hydrolyzed in 2 M HCl at 95°C for 2 hours, neutralized with an equal volume of 2 M NaOH, and centrifuged at 12,000*g* for 10 min. The liberated free-glycosyl units of the supernatant were determined using the glucose hexokinase kit (Wako, 298-65701) according to the manufacturer’s instructions.

### RNA analysis

Quantitative reverse transcription (RT)–PCR was performed as described previously (*48*). Briefly, total RNA was extracted from tissues using RNAiso Plus (Takara Bio). The purified RNA samples were then reverse-transcribed using the PrimeScript RT Reagent Kit with gDNA Eraser (Takara Bio). Real-time quantitative RT-PCR was performed using the ABI Prism Step-One system with a Reagent Kit from Takara Bio. Specific oligonucleotide primers for target gene sequences are listed in table S1. Arbitrary units of target mRNA were corrected to the expression of *36b4*.

### RNA-seq studies

Transcriptomics analyses were performed using RNA-seq as described previously (*52*). Total RNA was isolated from the entire GC muscle of FNIP1 mKO, WT FNIP1 Tg, FNIP1 S220A Tg, FNIP1 S220D Tg, and littermate control mice using RNAiso Plus (Takara Bio). RNA-seq using an Illumina HiSeq 4000 was performed by Beijing Novogene Bioinformatics Technology Co. Ltd. Two or three independent samples per group were analyzed. Paired-end and 150-nucleotide reads were obtained from the same sequencing lane. The sequencing reads were then aligned to the UCSC mm10 genome assembly using TopHat 2.0.14 with the default parameters. Fragments per kb of exon per million mapped reads were calculated using Cufflinks 2.2.1. The criteria for a regulated gene were a fold change of >1.5 (either direction) and a significant *P* value (<0.05) versus littermate controls. For pathway analysis, the filtered datasets were uploaded into DAVID Bioinformatics Resources 6.8 to review the biological pathways using the Functional Categories database. Gene Ontology analysis was used to interpret the data, and the regulated terms were ranked by *P* value. The RNA-seq data have been deposited in the National Genomics Data Center (NGDC) Genome Sequence Archive (GSA) and are accessible through GSA Series accession numbers CRA008211 (https://ngdc.cncb.ac.cn/search/?dbId=gsa&q=CRA008211), CRA011387 (https://ngdc.cncb.ac.cn/gsa/s/RdgAOIP6), and CRA013258 (https://ngdc.cncb.ac.cn/gsa/s/SrO1O3I7).

### Plasmid construction and site-directed mutagenesis

pEGFP-N1-FNIP1 (Addgene, #49175) was purchased from Addgene. The FNIP1 coding sequence was then subcloned and inserted into the pcDNA5 vector containing a Flag-tag at the N terminus. Site directed mutagenesis was performed using the QuikChange Kit (Stratagene) according to the manufacturer’s protocol: the amino acid S220, an FNIP1 phosphorylation site, was changed to Ala (A) or Asp (D), which mimicked dephosphorylation or phosphorylation, respectively. All constructs were confirmed by DNA sequencing.

### Primary cell culture

Primary muscle cells were isolated from the GC muscles of 4-week-old male mice as previously described (*31*). Briefly, GC muscles from both legs were removed. Minced tissue was digested in a collagenase/dispase/CaCl_2_ solution for 1.5 hours at 37°C in a shaking bath. Dulbecco’s modified Eagle’s medium (DMEM) supplemented with 10% fetal bovine serum (FBS) [Pre-plating medium (PPM)] was added, and samples were triturated gently before loading onto a Netwell filter (70 μm; BD Biosciences). The cell suspension was pelleted at 1000 rpm for 5 min. Cells were then resuspended in PPM and plated on an uncoated plate for differential plating. The cell suspension (not adherent) was centrifuged for 5 min at 1000 rpm, and the pellet was resuspended in growth medium (GM) [Ham’s F-10 medium supplemented with 20% FBS and basic fibroblast growth factor (2.5 ng/ml)]. Cells were plated on collagen coated flasks for expansion. Cells were fed daily with GM. For differentiation, cells were washed with PBS and refed with 2% horse serum/DMEM differentiation medium and refed daily. Cells were induced to differentiate for 3 days before various experiments.

### Cell transfection, RNA interference experiments, IP, and immunofluorescence

Transient transfections in HEK293T cells were performed using PEI Transfection Reagent (Polysciences) following the manufacturer’s protocol. siRNAs (GenePharma) targeting *Fnip1* (siRNA pool: #1, 5′-GCAGUUCACAGCAACCCAATT; #2, 5′-GGUGGCUACUGCUCAUCUUTT) were transfected into cells at a final concentration of 50 nM using Lipofectamine 2000 transfection reagent (Invitrogen) according to the manufacturer’s instructions.

Whole lysates from HEK293T cells 48 hours after transfection were used for CoIP studies. HEK293T cells were obtained from American Type Culture Collection were cultured at 37°C and 5% CO_2_ in DMEM supplemented with 10% FBS, penicillin (1000 U/ml), and streptomycin (100 g/ml). Cells were collected in lysis buffer [50 mM tris (pH 7.4), 150 mM NaCl, 1 mM EDTA, 1% Triton X-100, 1× cOmplete (Roche), 1 mM phenylmethylsulfonyl fluoride, 10 mM NaF, and 5 mM Na_3_VO_4_], and 1 μg of M2 anti-Flag (MilliporeSigma) antibody was incubated with extract and protein G–conjugated agarose beads. The immunoprecipitated proteins were analyzed by immunoblotting.

For immunofluorescence, HeLa cells were transfected with the indicated plasmids with Lipofectamine 2000 (Invitrogen). Thirty-six hours after transfection, the cells were washed with cold PBS and fixed with 4% paraformaldehyde in PBS at room temperature for 15 min. Then, the cells were permeabilized with 0.1% Triton X-100 and blocked with 10% goat serum at room temperature for 30 min, followed by incubation with primary antibody diluted in PBS containing 10% goat serum at 4°C overnight. The cells were then washed three times with PBS and incubated with fluorescence dye–conjugated secondary antibodies in PBS containing 10% goat serum for 1 hour at room temperature. Images were taken by stimulated emission depletion superresolved microscopy (Abberior Instruments).

### Antibodies and immunoblotting

Antibody directed against FLAG (F1804; 1:1000 dilution) was from Sigma-Aldrich; antibody directed against α-tubulin (bs1699; 1:5000 dilution) was from Bioworld; antibodies directed against FLCN (#3697S;1:1000 dilution), pTFEB (S122) (#86843; 1:1000 dilution), TFEB (#37785S; 1:1000 dilution), pAMPKα (T172) (#2535; 1:1000 dilution), AMPKα (#5831; 1:1000 dilution), phospho-Acetyl-CoA Carboxylase (pACC) (S79) (#11818; 1:1000 dilution), ACC (#3676; 1:1000 dilution), pS6K (T389) (#9234; 1:1000 dilution), and S6K (#2708; 1:1000 dilution) were from Cell Signaling Technology; antibodies directed against SDHA (14865-1-AP; 1:1000 dilution), ATP5A (14676-1-AP; 1:1000 dilution), UQCRC2 (14742-1-AP; 1:1000 dilution), and COX4 (11242-1-AP; 1:1000 dilution) were from ProteinTech; antibody directed against Tim23 (#611222; 1:1000 dilution) was from BD Biosciences; antibody directed against glyceraldehyde-3-phosphate dehydrogenase (GAPDH) (#2133; 1:3000 dilution) was from Signalway Antibody; antibody directed against FNIP2 (ab106611; 1:1000 dilution) was from Abcam, antibodies directed against FNIP1 (ab236547; 1:500 dilution) and pFNIP1 (S220) (1:200 dilution) were developed in the laboratory of Zhenji Gan with the help with Abcam. Specifically, after considering immunogenicity and uniqueness, C-QFCSPRRAF-pS-EQGP was chosen as the immunogen for the antibodies against pS220, and cystine was used for conjugation to the carrier protein. To generate a rabbit polyclonal antibody that recognizes FNIP1 pS220, the antigen peptide was injected into rabbits, and serum was collected and purified using an affinity column conjugated with unmodified peptides to exclude antibodies recognizing unmodified proteins and then subjected to an affinity column conjugated with the modified peptides to bind and purify the antibodies. The antibody was then eluted and concentrated. The specificity of the pFNIP1 (S220) antibody was evaluated by immunoblotting in the presence of blocking peptides. Western blotting studies were performed as previously described (*31*).

### Statistical analysis

All mouse and cell studies were analyzed by Student’s *t* test when two groups were compared. One-way analysis of variance (ANOVA) coupled to Fisher’s least significant difference (LSD) post hoc test was used when more than two groups were compared. Data represent the means ± SEM, with a statistically significant difference defined as a value of *P* < 0.05.
