# Supplementary material for: AMPK phosphorylation of FNIP1 (S220) controls mitochondrial function and muscle fuel utilization during exercise
Source: Sci Adv. 2024 Feb 7;10(6):eadj2752. doi: 10.1126/sciadv.adj2752 (PMC10849678; doi:10.1126/sciadv.adj2752)
Supplement: Supplementary file 1 — Figs. S1 to S5 Table S1 [file sciadv.adj2752_sm.pdf]

Science Advances
AAAS

# Supplementary Materials for

## AMPK phosphorylation of FNIP1 (S220) controls mitochondrial function and muscle fuel utilization during exercise

Liwei Xiao *et al.*

Corresponding author: Zhenji Gan, ganzj@nju.edu.cn; Xiaoduo Xie, xiexd8@mail.sysu.edu.cn

*Sci. Adv.* **10**, eadj2752 (2024)
DOI: 10.1126/sciadv.adj2752

**This PDF file includes:**

Figs. S1 to S5
Table S1

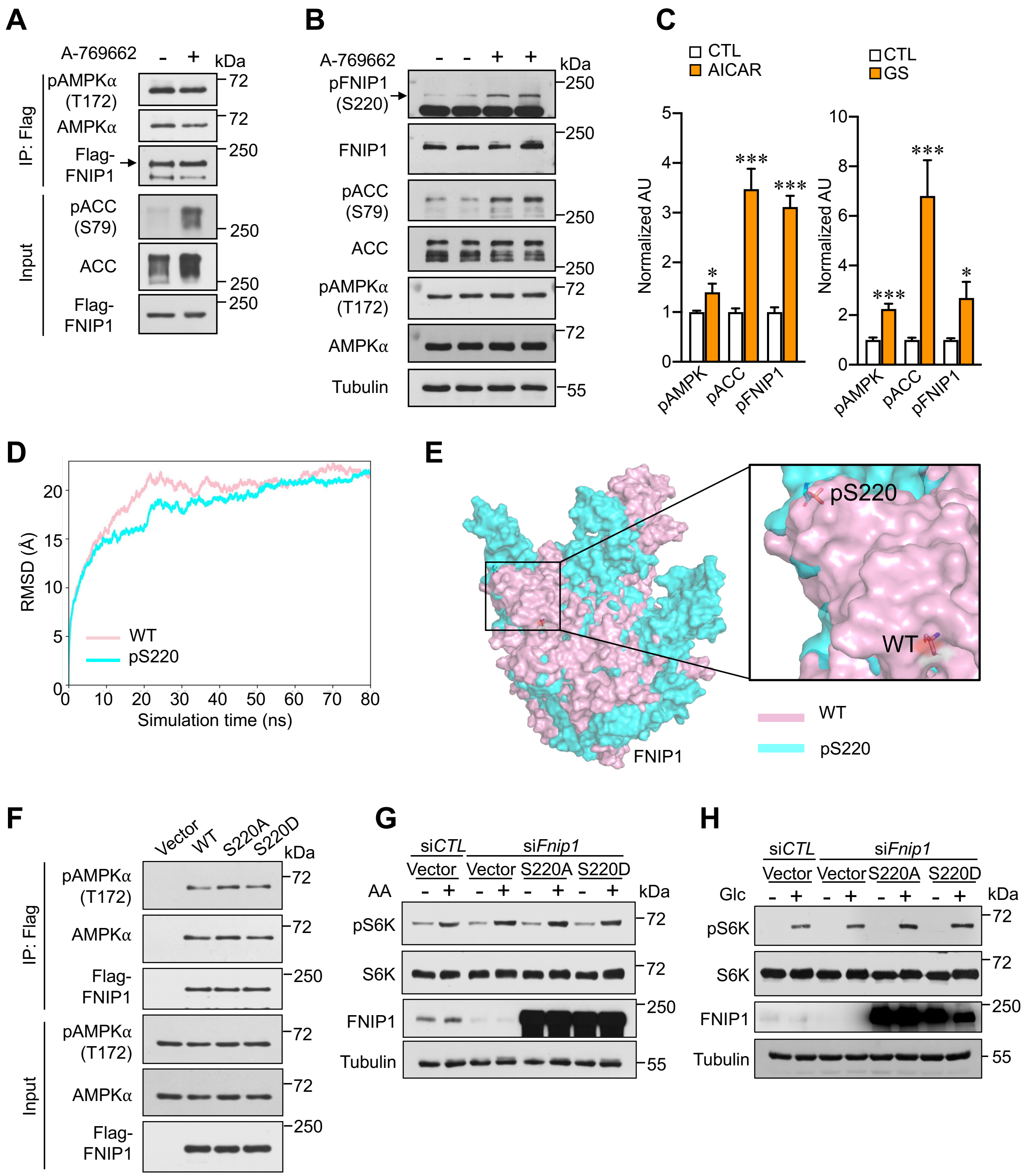


**Fig. S1 AMPK constitutively binds to and phosphorylates FNIP1 (S220).** (**A**) Effects of A-769662-induced AMPK activation on the interaction of FNIP1 and AMPK. Flag-FNIP1-transfected 293T cells were treated with vehicle or A-769662 (100 μM) for 1 hour, AMPK

activity was monitored by phosphorylation of Acetyl-CoA carboxylase (pACC), and interactions between AMPK and FNIP1 were detected by coimmunoprecipitation (CoIP) with the Flag antibody. (**B**) FNIP1 (S220) phosphorylation in response to A-769662. Primary myotubes were treated with A-769662 (300 μM, 1 hour), and cell lysates were immunoblotted with the indicated antibodies. (**C**) Quantification of pACC (S79), pAMPKα (T172) and pFNIP1 (S220) phosphorylation by signal ratios in Fig. 1F. CTL, control; GS, glucose starvation. (**D**) Molecular dynamic simulation analysis of FNIP1 in the wild-type (WT, pink) and S220 phosphorylation (pS220, green) states. (**E**) Overlap of WT and pS220 protein structural models. The predicted wild-type (WT, pink) and S220 phosphorylated (pS220, green) FNIP1 spatial structures were merged by AlphaFold2 and SWISS-MODEL algorithms. (**F**) The effect of FNIP1 phosphorylation on the AMPK-FNIP1 interaction. HEK293T cells transfected with Flag-FNIP1 variants were immunoprecipitated with Flag antibody and immunoblotted with the indicated antibodies. (**G** and **H**) The effects of FNIP1 phosphorylation on amino acid- or glucose-induced mTORC1 activity. FNIP1 was knocked down by siRNAs in HEK293T cells and then rescued by overexpressing Flag-FNIP1 variants. Cells were starved of amino acids (AA) for 50 min with or without 10 min of amino acid stimulation (G). Glucose starvation was performed by glucose (Glc) deprivation for 6 hours, and the cell lysates were analyzed by immunoblotting with the indicated antibodies (H). The experiments for A, B, F, G, and H were repeated at least 3 times. Error bars are shown as the mean ± SEM. *$P < 0.05$; ***$P < 0.001$. The $P$ value was determined by Student's t test.

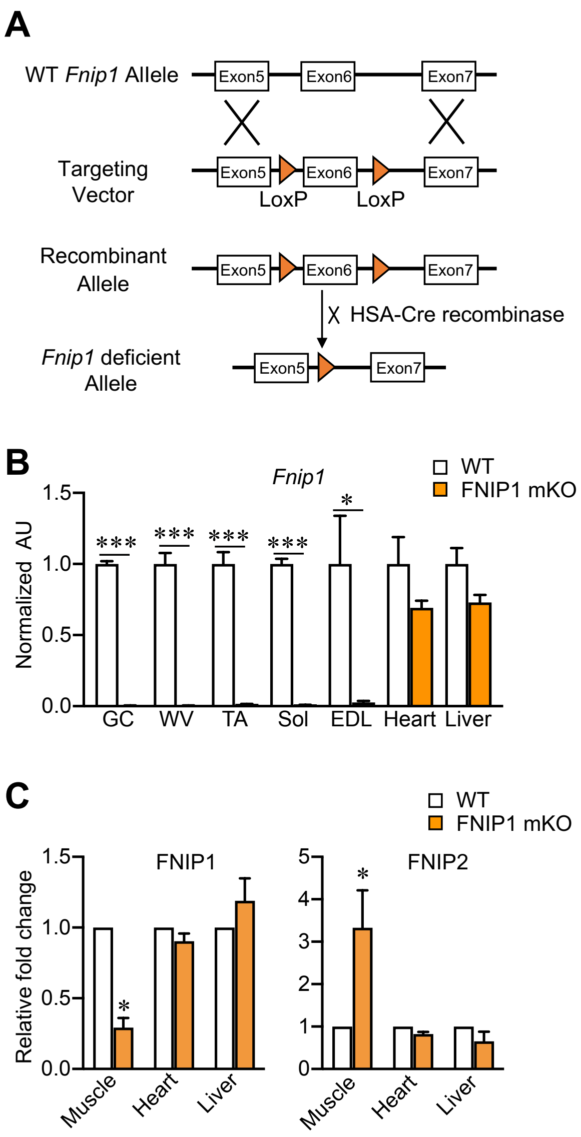


**Fig. S2 Generation of muscle-specific FNIP1 deletion mice.** (**A**) A schematic diagram depicting the generation of FNIP1 mKO mice. The *Fnip1* genomic allele was targeted by LoxP flanking Exon 6 and then crossed with HSA-Cre transgenic mice to remove Exon 6, leading to tissue-specific premature termination in the *Fnip1* exon 7 coding region. (**B**) *Fnip1* mRNA levels in tissue-specific gene-modified mice. RT–qPCR analysis of *Fnip1* expression in the gastrocnemius (GC), white vastus lateralis (WV), tibialis anterior (TA), soleus (Sol), extensor digital longus (EDL), heart, and liver of WT and FNIP1 mKO male mice, n = 3-4 mice per group.

(**C**) Quantification of FNIP1 and FNIP2 protein levels by signal ratios normalized to wild-type (WT) controls (=1.0). Immunoblotting analysis of WV muscle, heart and liver lysates of the indicated genotype using the indicated antibodies, n = 4-5 mice per group. Error bars are shown as the mean ± SEM. *$P < 0.05$; ***$P < 0.001$. The *P* value was determined by Student's t test.

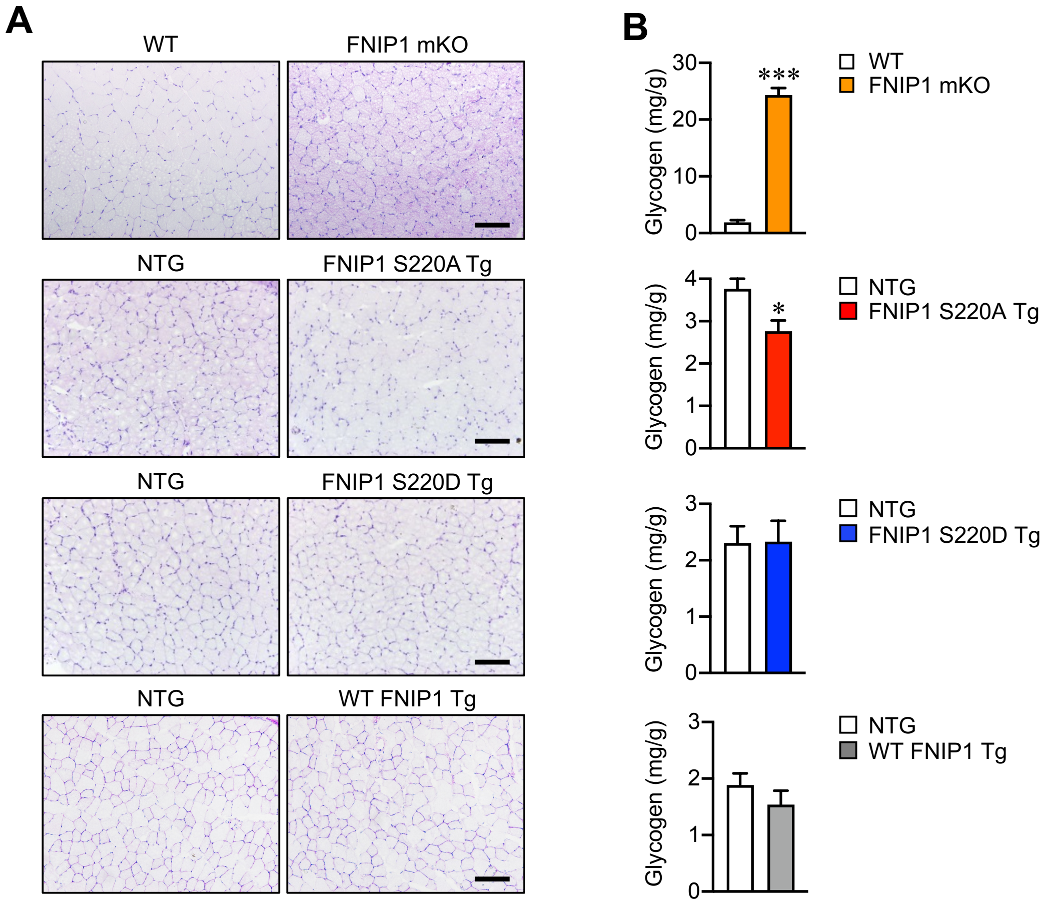


**C**

Genes upregulated in FNIP1 mKO muscle

| Go terms BP | Count | *P* value |
|---|---|---|
| immune system process | 75 | 1.10E-12 |
| angiogenesis | 47 | 7.90E-11 |
| lipid metabolic process | 85 | 8.30E-11 |
| inflammatory response | 57 | 1.40E-10 |
| cardiac muscle contraction | 18 | 6.20E-10 |
| cell migration | 43 | 1.20E-07 |
| fatty acid metabolic process | 32 | 2.20E-07 |
| antigen processing and presentation of exogenous peptide antigen via MHC class II | 11 | 3.80E-07 |
| antigen processing and presentation of exogenous peptide antigen via MHC class I | 8 | 4.10E-07 |
| regulation of blood pressure | 19 | 4.70E-07 |

**D**

Genes downregulated in FNIP1 mKO muscle

| Go terms KEGG | Count | *P* value |
|---|---|---|
| Spliceosome | 36 | 9.60E-08 |
| Oxytocin signaling pathway | 39 | 1.20E-07 |
| Glucagon signaling pathway | 30 | 2.70E-07 |
| Neurotrophin signaling pathway | 32 | 8.20E-07 |
| Colorectal cancer | 25 | 4.40E-06 |
| Insulin signaling pathway | 33 | 6.60E-06 |
| Gastric cancer | 34 | 1.30E-05 |
| Circadian rhythm | 14 | 1.40E-05 |
| Protein processing in endoplasmic reticulum | 37 | 1.80E-05 |
| TGF-beta signaling pathway | 25 | 2.20E-05 |

**Fig. S3 Glycogen content and metabolic gene expression in muscle tissues of FNIP1 mouse models.** (**A**) Muscle glycogen content in indicated mouse models. Periodic acid-Schiff (PAS) staining of cryosections from the indicated mice, scale bars: 100 μm, n = 4-8 mice per group. (**B**)

Enzymatic quantification of the tibialis anterior (TA) muscle glycogen content from the indicated mice, n = 6-9 mice per group. (**C** to **D**) Gene transcription analysis in FNIP1 mKO muscle. Gene Ontology (GO) enrichment analysis of gene transcripts upregulated (C) or downregulated (D) in FNIP1 mKO muscle, with the top ten terms shown. Error bars are shown as the mean ± SEM. **P* < 0.05; ****P* < 0.001. The *P* value was determined by Student's t test.

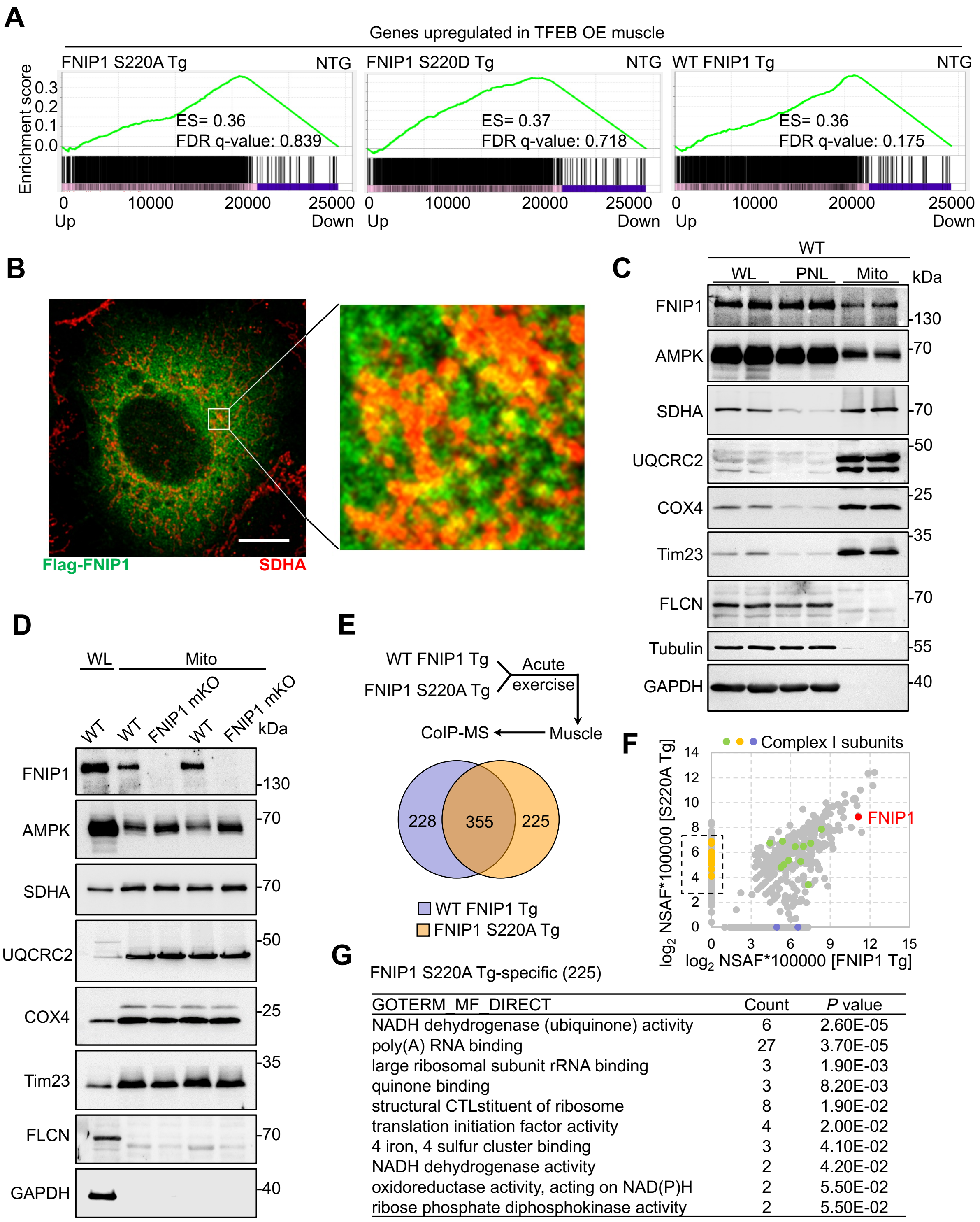


| GOTERM_MF_DIRECT | Count | *P* value |
|---|---|---|
| NADH dehydrogenase (ubiquinone) activity | 6 | 2.60E-05 |
| poly(A) RNA binding | 27 | 3.70E-05 |
| large ribosomal subunit rRNA binding | 3 | 1.90E-03 |
| quinone binding | 3 | 8.20E-03 |
| structural CTLstituent of ribosome | 8 | 1.90E-02 |
| translation initiation factor activity | 4 | 2.00E-02 |
| 4 iron, 4 sulfur cluster binding | 3 | 4.10E-02 |
| NADH dehydrogenase activity | 2 | 4.20E-02 |
| oxidoreductase activity, acting on NAD(P)H | 2 | 5.50E-02 |
| ribose phosphate diphosphokinase activity | 2 | 5.50E-02 |

**Fig. S4 The existence of the FNIP1 mitochondrial subpool with AMPK.** (**A**) Correlation of *Fnip1* transgenic and TFEB overexpression-induced gene expression in muscle tissues. GSEA of genes regulated in TFEB-overexpressing muscles in relation to genes altered in *Fnip1* transgenic

muscles. Genes regulated by FNIP1 S220A Tg (Left), FNIP1 S220D Tg (Middle), and WT FNIP1 Tg (Right) were ranked by fold difference and expressed on the x-axis. (**B**) Localization of FNIP1 and SDHA on mitochondria. HeLa cells were transfected with Flag-FNIP1 and costained with anti-Flag and anti-SDHA antibodies, and images were taken by stimulated emission depletion (STED) superresolved microscopy. Yellow indicates colocalized FNIP1 and SDHA. Scale bar, 10 μm. (**C** and **D**) Levels of the AMPK and FNIP1 proteins in the mitochondrial fraction. (C) Whole-tissue lysates (WL), postnuclear lysates (PNL), and the corresponding enriched mitochondrial fraction (Mito) from the sucrose gradient fractionation of mouse muscle were probed with antibodies against FNIP1, FLCN and AMPKα, with UQCRC2, SDHA, COX4, Tim23, Tubulin and GAPDH as fraction controls (n = 5). (D) Enriched mitochondrial fractions from muscle of FNIP1 mKO and WT littermate mice. n = 4 mice per group. (**E**) Identification of FNIP1 and FNIP1 (S220A)-interacting proteins in muscle tissues. Top: strategy for the identification of FNIP1-interacting proteins from WT FNIP1 and FNIP1 S220A transgenic muscle. Bottom: Venn diagram comparing different interacting proteins of WT FNIP1 Tg and FNIP1 S220A Tg muscles following acute running exercise. (**F**) Analysis of FNIP1 and FNIP1 (S220A)-interacting proteins in muscle tissues. The binding partners of FNIP1 and FNIP1 S220A were determined by mass spectrometry. Green, orange and blue: mitochondrial complex I subunits; red: FNIP1. (**G**) Gene Ontology (GO) enrichment analysis of FNIP1 S220A Tg-specific interacting proteins.

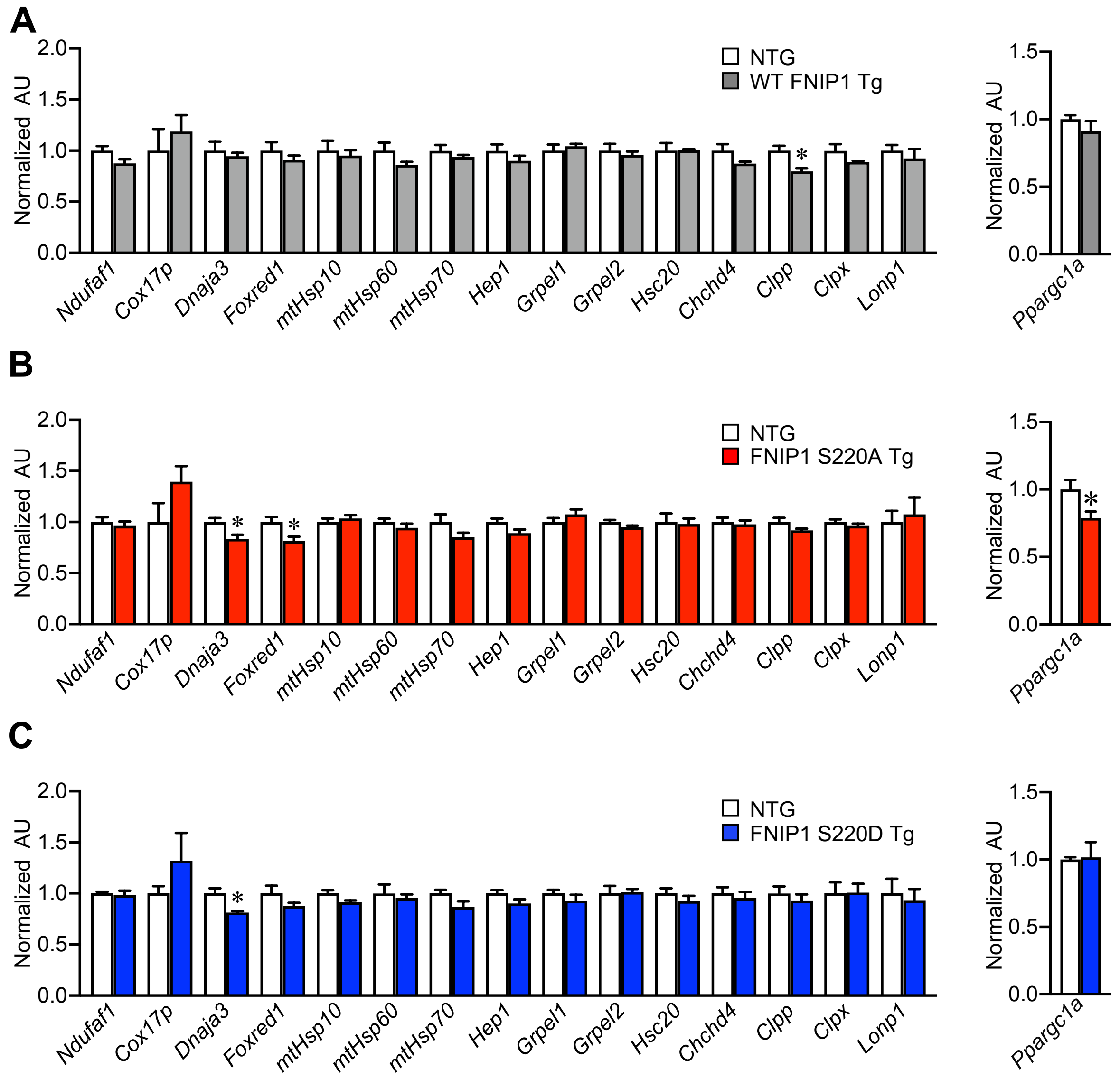


**Fig. S5 Mitochondria-related gene expression in *Fnip1* transgenic muscle.** (**A** to **C**) Mitochondrial chaperone genes and *Ppargc1a* mRNA levels in *Fnip1* transgenic muscle. RT–qPCR analysis of mitochondrial chaperone gene (left) and *Ppargc1a* (right) mRNA levels in GC muscle tissues from WT FNIP1 Tg (A), S220A Tg (B), and S220D Tg (C) transgenic mice compared to NTG controls, n = 5-9 mice per group. Error bars are shown as the mean ± SEM. *$P$ < 0.05. The $P$ value was determined by Student's t test.

## Table S1

## RT-qPCR primers

| **Supplementary Table 1. RT-qPCR primers** | | |
|---|---|---|
| ***Mouse Gene*** | ***Forward*** | ***Reverse*** |
| *36b4* | *5'-ATCCCTGACGCACCGCCGTGA* | *5'-TGCATCTGCTTGGAGCCCACGTT* |
| *Chchd4* | *5'-AAACTCCTAGCAGTGCCGAGCT* | *5'-CAGGAGAAGGCAGACTTGAACTG* |
| *Clpp* | *5'-TGGGCCCGATTGACGACAGTG* | *5'-TAGATGGCCAGGCCCGCAGT* |
| *Clpx* | *5'-CCAGGCTGGATATGTAGGTGAAG* | *5'-TGAATGCCTGGCACACTGCCAA* |
| *Cox17p* | *5'-AGGAGAACGGCAAGCTTCAA* | *5'-TCACACAGCAGACCACCATT* |
| *Dnaja3* | *5'-TATGCCGAGGATGAGACTGACG* | *5'-CTCTCGCCTATCCTTGCTTCCT* |
| *Fnip1* | *5'-AGTAATGGGCTGCTTGGAAA* | *5'-CAAAGAAAGAGGCACTCCTGA* |
| *Foxred1* | *5'-GAAGGAGCCAAAGTCTGCCTGA* | *5'-GAGCAAAGACCAGGCATCAAACC* |
| *Grpel1* | *5'-GAATCTACGGCAGAGAAGCCAG* | *5'-GGTTGCCTTCTCCAGGATGTCT* |
| *Grpel2* | *5'-GAACCCAGAGATGTGTGGAAGAC* | *5'-ACAGCACTTGGCAGTCTTCTCC* |
| *Hep1* | *5'-GTGGAAGCGGACCACTATCAAC* | *5'-AACCAGGGCAAGTCACGATGAC* |
| *Hsc20* | *5'-CACTCGTGACTACTTCAGCCTC* | *5'-GGCTGAAGAAATCTGGGTGGAC* |
| *Lonp1* | *5'-CATTGCCTTGAACCCTCTC* | *5'-ATGTCGCTCAGGTAGATGG* |
| *mtHsp10* | *5'-GCCGAAACTGTAACCAAAGGTGG* | *5'-CTCCAACTTTCACACTGACAGGC* |
| *mtHsp60* | *5'-TGCTCATCGGAAGCCATTGGTC* | *5'-TTGACTGCCACAACCTGAAGACC* |
| *mtHsp70* | *5'-GTTGGTATGCCAGCAAAACGGC* | *5'-CAAGCATCACCATTGGAGGCAC* |
| *Ndufaf1* | *5'-GTGCCATGATCTCCAGGATTCC* | *5'-GGATAAACTCCGTGTCTTGCCTG* |
| *Ppargc1a* | *5'-GGACATGTGCAGCCAAGACTCT* | *5'-CACTTCAATCCACCCAGAAAGCT* |
